# Pyrroline-5-Carboxylate Reductase 1: a novel target for sensitizing multiple myeloma cells to bortezomib by inhibition of PRAS40-mediated protein synthesis

**DOI:** 10.1186/s13046-022-02250-3

**Published:** 2022-02-01

**Authors:** Inge Oudaert, Hatice Satilmis, Philip Vlummens, Wouter De Brouwer, Anke Maes, Dirk Hose, Elke De Bruyne, Bart Ghesquière, Karin Vanderkerken, Kim De Veirman, Eline Menu

**Affiliations:** 1 Department of Hematology and Immunology-Myeloma Center Brussels, Vrije Universiteit Brussel, Laarbeeklaan 103, 1090 Jette Brussels, Belgium; 2 Department of Clinical Hematology, Ghent University Hospital, Gent, Belgium; 3 Department of Hematology, Vrije Universiteit Brussel, Universitair Ziekenhuis Brussel, Laarbeeklaan 101, 1090 Jette Brussels, Belgium; 4 Metabolomics Expertise Center, VIB Center for Cancer Biology (VIB-CCB) - Department of Oncology, KU Leuven, Leuven, Belgium

**Keywords:** Multiple myeloma, Proline, PYCR1, Hypoxia, Protein synthesis

## Abstract

**Background:**

Multiple myeloma (MM) remains an incurable cancer despite advances in therapy. Therefore, the search for new targets is still essential to uncover potential treatment strategies. Metabolic changes, induced by the hypoxic bone marrow, contribute to both MM cell survival and drug resistance. Pyrroline-5-carboxylate reductase 1 and 2 (PYCR1 and PYCR2) are two mitochondrial enzymes that facilitate the last step in the glutamine-to-proline conversion. Overexpression of PYCR1 is involved in progression of several cancers, however, its’ role in hematological cancers is unknown. In this study, we investigated whether PYCR affects MM viability, proliferation and response to bortezomib.

**Methods:**

Correlation of PYCR1/2 with overall survival was investigated in the MMRF CoMMpass trial (653 patients). OPM-2 and RPMI-8226 MM cell lines were used to perform in vitro experiments. RPMI-8226 cells were supplemented with ^13^C-glutamine for 48 h in both normoxia and hypoxia (< 1% O_2_, by chamber) to perform a tracer study. PYCR1 was inhibited by siRNA or the small molecule inhibitor pargyline. Apoptosis was measured using Annexin V and 7-AAD staining, viability by CellTiterGlo assay and proliferation by BrdU incorporation. Differential protein expression was evaluated using Western Blot. The SUnSET method was used to measure protein synthesis. All in vitro experiments were performed in hypoxic conditions.

**Results:**

We found that PYCR1 and PYCR2 mRNA expression correlated with an inferior overall survival. MM cells from relapsed/refractory patients express significantly higher levels of PYCR1 mRNA. In line with the strong expression of PYCR1, we performed a tracer study in RPMI-8226 cells, which revealed an increased conversion of ^13^C-glutamine to proline in hypoxia. PYCR1 inhibition reduced MM viability and proliferation and increased apoptosis. Mechanistically, we found that PYCR1 silencing reduced protein levels of p-PRAS40, p-mTOR, p-p70, p-S6, p-4EBP1 and p-eIF4E levels, suggesting a decrease in protein synthesis, which we also confirmed in vitro. Pargyline and siPYCR1 increased bortezomib-mediated apoptosis. Finally, combination therapy of pargyline with bortezomib reduced viability in CD138^+^ MM cells and reduced tumor burden in the murine 5TGM1 model compared to single agents.

**Conclusions:**

This study identifies PYCR1 as a novel target in bortezomib-based combination therapies for MM.

**Supplementary Information:**

The online version contains supplementary material available at 10.1186/s13046-022-02250-3.

## Background

Multiple myeloma (MM) is the second most common hematological cancer, characterized by the accumulation of monoclonal plasma cells in the bone marrow (BM) [1]. Despite major therapeutic advances, myeloma remains an incurable cancer due to accumulating toxicities and evolvement of functional drug resistance. One of the players herein is the BM microenvironment with its hypoxic nature, leading to metabolic changes that stimulate cancer progression [2–5]. Current therapies for myeloma include combination therapy of several drugs such as the proteasome inhibitor bortezomib, the immunomodulating agent lenalidomide and steroid dexamethasone, followed by autologous stem cell transplantation if possible [6]. Recently, immune therapy has also been approved for MM patients with the monoclonal antibody daratumumab against CD38 showing promising results [7]. Bortezomib remains a key drug in the treatment of myeloma since its FDA approval in 2003. Both newly diagnosed and relapsed/refractory patients are treated with this proteasome inhibitor, which generates high response rates [8].

The ability to rapidly increase protein synthesis is an important factor for cancerous growth. The PRAS40 pathway or proline-rich Akt substrate of 40 kDa, was first discovered in 2003 by Kovacina et al. [9]. PRAS40 is involved in several biological processes, including protein synthesis, apoptosis, growth, autophagy and oxidative stress [10, 11]. The PRAS40 pathway is regulated by PI3K/Akt signaling pathway and affects several signaling pathways, such as mammalian target rapamycin (mTOR), pyruvate kinase M2 (PKM2) and nuclear factor kappa-B (NF-κB) [10].

Cancer cells are also known for their adaption mechanisms to accommodate for their own survival. In a hypoxic environment, cancer cells prefer to perform aerobic glycolysis rather than oxidative phosphorylation to generate energy, whereby more glucose is being transformed into lactate instead of pyruvate, a process referred to as the Warburg effect [12, 13]. In MM, some hypoxic alterations have been associated with drug resistance: a hypoxia-induced increase in hexokinase II was accompanied by autophagy stimulation, a process used by cancer cells to avoid drug-mediated cell death [14]. Maiso et al. showed that targeting lactate dehydrogenase A (LDHA) and hypoxia-inducible factor alpha (HIF1α) resensitized myeloma to bortezomib and could impair tumor growth [15]. Also, gene expression profiling of primary patient samples revealed that chronic hypoxia increased expression of several glycolytic enzymes, as well as H3K9 demethylases such as histone lysine demethylase 3A (KDM3A) which were linked to resistance and survival [16]. Furthermore, hypoxia has been shown to stimulate glutamine metabolism in several cancers such as lung cancer, neuroblastoma and pancreatic cancer [17–19]. Also in myeloma, cancer cells are highly addicted to glutamine metabolism, underscoring its’ importance in the BM milieu [20].

On the other hand, proline metabolism has been implemented in tumorigenesis, as cancer cells tend to convert more glutamine to proline compared to normal cells [21]. Moreover, proline can be used by cancer cells as energy source and/or as precursor of protein synthesis [22]. The conversion of glutamine to proline consists of several steps. In the final conversion step, pyrroline-5-carboxylate is transformed into proline, a reaction catalyzed by pyrroline-5-carboxylate reductase (PYCR). Three PYCR enzymes have been identified; PYCR1, PYCR2 and PYCR3 (or PYCRL). PYCR1 and PYCR2 are present in the mitochondria and show a 84% sequence homology. PYCR3 is mainly found in the cytoplasm, and converts ornithine to proline. PYCR3 shows only 45% similarity in amino acids to the other PYCR enzymes [23–25].

So far, it is not known whether proline metabolism is increased in hypoxia, despite the fact that some studies have revealed the potential of PYCR1 as a promising target in cancer therapy. For instance, patients with non-small cell lung cancer (NSCLC) show an overexpression of PYCR1, which is associated with poor survival. PYCR1 knockdown resulted in inhibition of colony formation, reduced proliferation and induced cell cycle arrest in vitro [26]. In breast cancer, PYCR1 expression was associated with tumor size, grade, invasiveness and survival. In vitro experiments using small-hairpin RNA reduced growth and invasiveness, but also enhanced the cytotoxic effects of doxorubicin in both estrogen receptor (ER) positive and ER negative breast cancer cell lines [27]. PYCR1 has also been investigated in hepatocellular cancer, where PYCR1 interference reduced cell growth and survival both in vitro and in vivo [28]. However, the role of PYCR1 in hematological malignancies is currently unknown.

In this study, we aimed to evaluate the impact of hypoxia on proline metabolism and whether this pathway plays a role in myeloma viability and drug resistance. We investigated the association of PYCR1/2 expression with survival in the MMRF CoMMpass trial. We next inhibited PYCR expression under hypoxia by siRNA and a small molecule inhibitor, to study the impact on protein synthesis, proliferation, apoptosis and response to bortezomib. Proteome profiling was performed to identify the pathway of action. Finally, we investigated whether the PYCR1 inhibitor could increase bortezomib efficacy in vivo.

## Methods

### Cell culture

The human MM cell lines (HMCL) OPM-2, LP-1, JJN-3, ANBL-6 and RPMI-8226 and human stromal cell line HS-5 were obtained from ATCC (Molsheim, France). The identity of the cell lines was regularly checked by short-tandem repeat analysis. Cell lines were regularly tested for mycoplasma contamination and passaged no more than one month prior to experiments. HMCL were cultured in RPMI-1640 medium (Thermo Fisher Scientific, Aalst, Belgium) supplemented with 10% fetal bovine serum (FBS) (Hycone, Logan, UT, USA), 2 mM L-glutamine (Thermo Fisher Scientific) and 100 units/ml penicillin/streptomycin (Thermo Fisher Scientific) at 37 °C in 5% CO_2_. Additionally, ANBL-6 cells were cultured in presence of 2 ng/mL recombinant IL-6 (R&D Systems, Oxon, UK). HS-5 cell line was cultured in DMEM medium supplemented with 10% FBS, 2 mM L-glutamine, 1 mM sodium pyruvate, 1% MEM NEAA (Thermo Fisher Scientific) and 100 units/ml penicillin/streptomycin.

### Drugs and reagents

Bortezomib was purchased from Selleckchem (Munich, Germany) and dissolved in dimethylsulfoxide at a stock concentration of 10 mM. For the final concentration, we chose concentrations that induced 20% cell death: for the OPM2 this was 2 nM while for RPMI-8226 this was 2.5 nM. SiRNA was purchased from Qiagen (Antwerp, Belgium) and dissolved in sterile RNAse free water at a stock concentration of 10 µM. The gene-specific sequences were as followed: PYCR1: sense (5’-CAC GGG AGC UGC AGU CCA UTT-3’), antisense (5’-AUG GAC UGCAGC UCC CGU GTG-3’), PYCR2: sense (5’GGA UUA GAC UGG GUU UAU ATT-3’), antisense (5’ UAU AAA CCC AGU CUA AUC CTG-3’). AllStars Negative Control siRNA (Catalog #1,027,280, Qiagen) was used as transfection control. SiRNA for PYCR1 was used at 20 nM final concentration, while PYCR2 knockdown was established using 50 nM as final concentration. SiRNA and Lipofectamine 2000 (Thermo Fisher Scientific) were mixed together in Opti-MEM Reduced Serum Medium (Gibco), incubated for 15 min at room temperature and added dropwise to the cells. Pargyline was dissolved in proline-free RPMI-1640 medium (US Biologicals, MA, USA) at a stock concentration of 250 mM.

### Hypoxic culture

For experiments, HMCL were incubated at indicated times in a hypoxia chamber (STEMCELL™ technologies, Grenoble, France) containing a nitrogen/carbon dioxide-balanced gas with 1% of oxygen [29].

### Analysis of PYCR gene expression levels

The MMRF CoMMpass Trial (NCT01454297) is a longitudinal study of newly diagnosed MM patients in which genomic data is collected at diagnosis and subsequent relapse(s). The sequencing and clinical data, including survival information, are publicly available through the MMRF research gateway portal (https://research.themmrf.org) [30]. We used the Interim Analysis 12 data which consists of 653 patient BM samples taken at diagnosis for which both survival data and RNA expression data are available. We correlated PYCR1/2 gene expression levels with survival information to analyze the prognostic value of PYCR using the MaxStat R package as previously described [31].

### Tracer study

RPMI-8226 cells were seeded at 200,000 cells/ml in RPMI-1640 medium, supplemented with 10% FBS, 2 mM L-glutamine, 100 units/ml penicillin/streptomycin, 10 mM ^13^C-glutamine (provided by Metabolomics Expertise Center (MEC), VIB-KU Leuven, Belgium) and cultured in normoxia (21% oxygen) and hypoxia (1% oxygen). After 48 h, cells were isolated, centrifuged and washed with ice cold 0.9% NaCl solution. Afterwards, cells were centrifuged again and extraction buffer (80% methanol) was added during 2–3 min on ice. Extraction mix was transferred to eppendorf on ice and centrifuged at 20,000 g during 10 min at 4 °C. Supernatant was isolated and put on dry ice and shipped to MEC for analysis. All results are shown relative to cell number.

### Viability and apoptosis assays

HMCL were seeded at 250,000 cells/ml in proline-free RPMI-1640 medium with 10% FBS, supplemented with 2 mM L-glutamine, 100 units/ml penicillin/streptomycin and 2 mM HEPES (additional buffer for hypoxic culture, Thermo Fisher Scientific). For siRNA experiments, cells were seeded at 200,000 cells/ml. Viability was measured by CellTiter Glo Luminescent Cell Viability Assay (Promega, Madison, WI, USA) according to the manufacturer’s instructions. To measure apoptosis, cells were harvested and washed with FACS flow, centrifuged and stained with 98 µl of 1 × binding buffer, 1 µl of Annexin V-APC (BD Biosciences, Erembodegem, Belgium) and 1 µl of 7-AAD (BD Biosciences). After 15 min of incubation in the dark, samples were analysed by flow cytometry using FACSCanto flow cytometer (BD Biosciences). All experiments were performed in hypoxic conditions.

### BrdU stainings

HMCL were seeded at 200,000 cells/ml and treated with siRNA for 48 h (RPMI-8226) or 72 h (OPM-2). Afterwards, cells were treated with 1 mg/ml Bromodeoxyuridine (BrdU) for 4 h. Cells were isolated, washed with FACS flow, and stained for 10 min with paraformaldehyde at 4 °C. The cells were incubated overnight in PBS (Gibco) 0.2% tween (Sigma-Aldrich, Diegem, Belgium) at room temperature, washed with FACS flow, stained for 30 min with 2 M HCl and washed with both FACS flow and a mixture of PBS + 0.5% Triton + 10% serum. Cells were then treated with 3 µl of Anti-BrdU-Fluorescein (Catalog #1120269001, Sigma-Aldrich) in 50 µl of PBS + 0.5% Triton (Sigma-Aldrich) + 10% FBS and incubated in the dark for 30 min. After a next wash step, FITC positivity was measured by flow cytometry on a FACSCanto flow cytometer. All experiments were performed in hypoxic conditions.

### Quantitative real-time PCR

Total RNA was extracted and purified using the NucleoSpin RNA plus kit (Macherey–Nagel, Düren, Germany) and 1 µg of RNA was converted to cDNA by the Verso cDNA Synthesis Kit (Thermo Fisher Scientific) according to the manufacturer’s protocol. Real-time PCR was performed using SYBR Green (PowerUp SYBR™ Green Master Mix, Applied Biosystems, Thermo Fisher Scientific) in a final volume of 25 µL, consisting of 1 µL of cDNA, 12.5 µL of SYBR Green, 1 µL of Primer Mix 10 pmol/μl and 10.5 µL of nuclease-free water. The expression level of mRNA was quantified by qRT-PCR using the QuantStudio 12 K Flex Real-Time PCR System (Thermo Fisher Scientific). The housekeeping genes ABL and β-ACTIN were used for data normalization. Gene-specific primer sequences were as follows: PYCR1: forward (5’-GTG GTT ACT GTG GGT GGA ATA-3’), reverse (5’-CAG ATG CCC TCC AAG ATG TG-3’), PYCR2: forward (5’-TGC AAG CCA GAC ACA TCG TGG T-3’), reverse (5’-CTG TTG CTC ATG CAG CGA ATC AC-3’), ABL: forward (5’-GAG GGC GTG TGG AAG AAA TA-3’), reverse (5’-CACAG GTT AGG GTG TTT GA-3’), beta-ACTIN: forward (5’-CAC TCT TCC AGC CTT CCT TC-3’), reverse (5’-GTA CAG GTC TTT GCG GAT GT-3’). All primers were purchased from Integrated DNA technologies (Leuven, Belgium). The -dCt values are portrayed when evaluating the basal expression levels of PYCR genes in MM and stromal cell lines. The comparative 2^−ΔΔCt^ method is used to investigate differential expression compared to a control sample.

### Proteome profiling

We identified the underlying pathway of PYCR1 inhibition with a Human Phospho-Kinase Array Kit (R&D Systems, UK, #ARY003B) according to the manufacturer’s instructions. Chemiluminescence was visualized and analyzed using Li-Cor Odyssey Fc (Li-COR Biosciences, Bad Homburg, Germany). Quantification was performed using Image Studio Lite version 5.2 software.

### Western blot analysis

HMCL were seeded in a 6-well plate and cultured during 72 h or 96 h in proline-free RPMI medium with 10% FBS, 2 mM L-glutamine, 2 mM HEPES and siRNA. All experiments were performed in hypoxic conditions. Cells were lysed in lysis buffer containing 50 mM Tris, 150 mM NaCl, 1% Nonidet P40, and 0.25% sodium deoxycholate. The following protease and phosphatase inhibitors were added: 4 mM Na_3_VO_4_, 1 mM Na_4_P_2_O_7_, 2 µg/mL aprotinin, 50 µg/mL leupeptin, 500 µg/mL trypsin inhibitor, 10 µM benzamidine, 2.5 mM pnp benzoate (all from Sigma-Aldrich), 50 mM NaF, 5 mM ethylenediaminetetraacetic acid (both from VWR International), 1 mM 4-(2-aminoethyl) benzenesulfonyl fluoride hydrochloride, and 50 µg/mL pepstatin A (both from ICN). Western blot analysis on these cell lysates was performed as previously described [13]. Chemiluminescence was visualized and analysed using Li-Cor Odyssey Fc (Bad Homburg, Germany). Quantification was performed using Image Studio Lite version 5.2 software. Antibodies used for analysis were: β-ACTIN (#4967), PARP (#9542), Caspase 3 (#9665), α-tubulin (#2144), p-AKT (#4065), AKT (#9272), p-p42/44 MAPK (#9106), p42/44 MAPK (#9102), c-MYC (#5605), p-p70 (#9234), p70 (#2708), p-PRAS40 (#13,175), PRAS40 (# 2691), p-S6 (#4858), S6 (#2217), p-4EBP1 (#2855), 4EBP1 (#9644), p-eIF4E (#9741), eIF4E (#2067), p-SAPK/JNK (#9255), SAPK/JNK (#9252) and CHOP (#2895) all from Cell Signaling Technology (Leiden, The Netherlands). PYCR1 (ab206693) and PYCR2 (ab103535) were purchased from Abcam (Cambridge, UK). IRE1a (sc-20790) was purchased from Santa Cruz Biotechnology. The horseradish peroxidase (HRP)-coupled anti-rabbit (#7074) and anti-mouse (#7076) antibodies were purchased from Cell Signaling Technology.

### Isolation of CD138 + and CD138- Cells from bone marrow samples

BM samples were collected as part of routine diagnostic or evaluation purposes after patients’ written informed consent and in accordance with the Declaration of Helsinki and institutional research board approval from Brussels University Hospital (B.U.N. 143201838414). Mononuclear cells were isolated by density gradient centrifugation with Lymphoprep™ (STEMCELL™ technologies, Grenoble, France). CD138^+^ cells were isolated by magnetic activated cell sorting using human CD138 MicroBeads (Miltenyi Biotec, Gladbach, Germany) according to manufacturer’s instructions.

### Proline assay

Proline content was measured in cell lysates using Proline Assay kit (ARG82030, Arigo biolaboratories, TE HUISSEN, The Netherlands). Briefly, cells were collected and centrifuged at 1500 rpm for 5 min, washed with PBS, and dissolved in 70 µl of assay buffer. Samples were sonicated according to the manufacturer’s instructions. After 1 h of incubation at 90 °C in the dark, OD values were measured using an microplate reader (Bio-RAD, Brussels, Belgium) at 595 nm. All experiments were performed in hypoxic conditions.

### Puromycin uptake

Cells were cultured in 6 well plates with the appropriate compounds during 48 h or 72 h as described earlier. After treatment, cells were incubated with 1 µM of puromycin (Cat nr S7417, Selleckchem) during 30 min, followed by pellet isolation. Cells were lysed in lysis buffer (see earlier), and western blot was performed to measure puromycin uptake. We used the anti-puromycin antibody (#MABE343, Sigma-Aldrich) for analysis. All experiments were performed in hypoxic conditions.

### Murine experiment

C57BL/KalwRij mice were purchased from Envigo Laboratories (Horst, The Netherlands). They were housed and treated following conditions approved by the Ethical Committee for Animal Experiments of the Vrije Universiteit Brussel (licence No LA1230281, CEP No 20–281-6). On day 0, all mice (*n* = 9/group) were inoculated (I.V.) with 1 million of eGFP positive 5TGM1 cells dissolved in 200 µl of RPMI-1640 medium. Mice were treated intraperitonealy with 100 mpk pargyline 5x/week, and 0.6 mpk bortezomib intraperitonealy 2x/week starting 14 days after inoculation. After 30 days, vehicle mice reached end-stage and all mice were sacrificed. We isolated BM, followed by red blood cell lysis. Tumor burden in the BM was assessed by eGFP positivity on flow cytometry. For western blot, we further purified the 5TGM1 cells by negative selection (CD11b-) using CD11b MACS beads (Miltenyi Biotec) according to the manufacturer’s instructions.

During the experiment, the general health of all mice was evaluated daily, and body weight was measured 2x/week.

### Statistical Analysis

Results were analysed with GraphPad Prism 8.0 software (GraphPad Software Inc, La Jolla, CA, USA). All data represent the mean ± standard deviation (SD), and results were analysed using the Mann–Whitney U test (*n* = 3), Wilcoxon test, Kruskal–Wallis test (*n* = 3) or one-way ANOVA (n ≥ 5). *p* ≤ 0.05 (*), *p* ≤ 0.01 (**) and *p* ≤ 0.001 (***) and *p* ≤ 0.0001 (****) were considered statistically significant.

## Results

### PYCR1 and PYCR2 overexpression is associated with adverse overall survival in myeloma patients

**Fig. 1:**
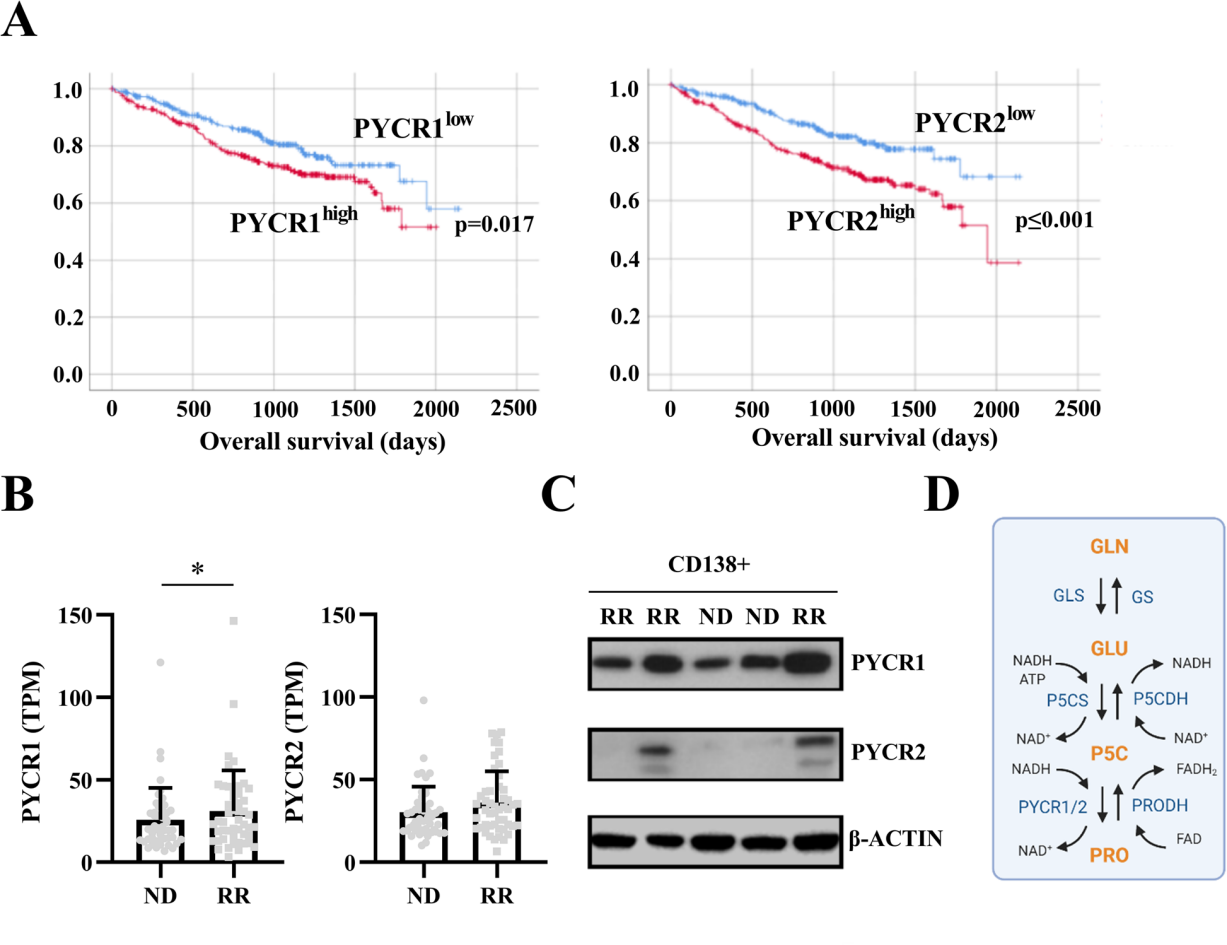
PYCR1 and PYCR2 correlate with lower overall survival in MM patients. **A** Survival curves for MM patients with high (red line) or low (blue line) PYCR1 or PYCR2 RNA expression (MMRF CoMMpass trial, *n* = 653). **B** PYCR1 and PYCR2 mRNA expression levels of samples taken at first diagnosis (ND, *n* = 49) and relapse/refractory (RR, *n* = 49) stage (MMRF CoMMpass trial). **C** Basal PYCR1 and PYCR2 expression in the CD138^+^ fraction of primary patient samples on protein level (*n* = 5). **D)** Overview of the glutamine-to-proline conversion. Created with BioRender.com. GLN = glutamine, GLU = glucose, P5C = pyrroline-5-carboxylate, PRO = proline, GS = glutamine synthase, GLS = glutaminase, P5CDH = pyrroline-5-carboxylate dehydrogenase, P5CS = pyrroline-5-carboxylate synthase, PRODH = proline dehydrogenase, PYCR = pyrroline-5-carboxylate reductase, TPM = transcripts per million, ND = newly diagnosed, RR = relapsed/refractory. Significance was determined by two-tailed Wilcoxon test. * *p* ≤ 0.05

Assessing the CoMMpass trial, we found that both high mRNA expression of PYCR1 and PYCR2 is associated with a significantly shorter overall survival (Fig. 1A). PYCR1 mRNA expression increased during progression as relapsed/refractory samples expressed higher levels of PYCR1 than samples isolated from the same patients when first diagnosed (Fig. 1B, longitudinal samples). PYCR2 expression did not significantly change after relapse (Fig. 1B). We next validated PYCR1/2 expression on protein level (Fig. 1C). Indeed, all assessed samples (*n* = 5, CD138^+^ MM cells) expressed PYCR1 on protein level, while we were able to detect PYCR2 expression in only two out of five patients. Again, the highest PYCR1 protein expression was found in relapsed/refractory samples. Patient and disease characteristics for these data are shown in Suppl table 1. An overview of the PYCR pathway and its involving enzymes is shown in Fig. 1D.

### Glutamine-to-proline conversion is upregulated in a hypoxic environment

**Fig. 2:**
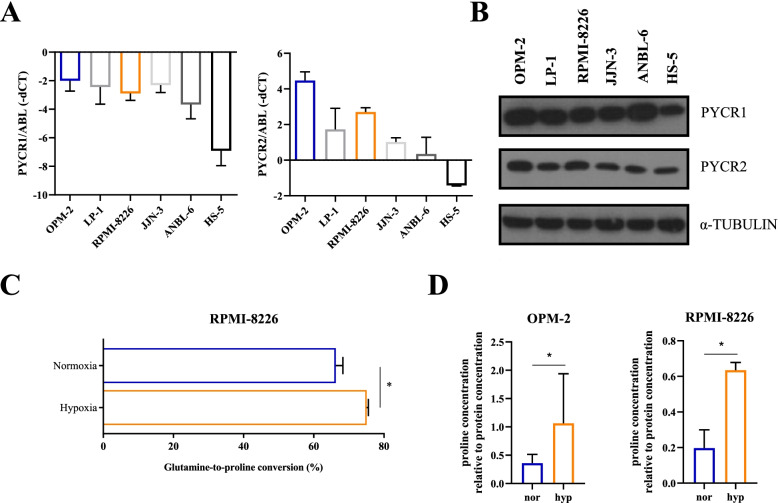
Hypoxia increases proline production in vitro*.* **A**, **B** Basal expression of PYCR1 and PYCR2 in 5 human MM cell lines (OPM-2, LP-1, RPMI-8226, JJN-3 and ANBL-6) and one stromal cell line (HS-5) is shown on RNA (**A**) and protein level (**B**) in normoxic conditions (*n* = 3). **C** Results of a tracer study where ^13^C-labeled glutamine was added to RPMI-8226 during 48 h in normoxic and hypoxic conditions (*n* = 1, measured in triplicate). The conversion rates of ^13^C-glutamine to proline are measured and shown in percentage. **D** Change in proline concentration, measured by ELISA after 48 h of culture in normoxic and hypoxic conditions (*n* = 3). nor = normoxia, hyp = hypoxia. Significance was determined by Mann–Whitney U test. * *p* ≤ 0.05

To investigate the role of PYCR enzymes on MM viability in vitro, we measured the RNA and protein expression of both enzymes on five HMCL originating from different MM subclasses (MMSET: OPM-2, MMSET/FGFR3: LP-1, cMAF: RPMI-8226, JJN-3 and IL-6 dependent cell line ANBL-6) and one stromal cell line HS-5 (Fig. 2A-B). The MM cell line OPM-2 showed the highest PYCR1 and PYCR2 expression, while the stromal cell line HS-5 showed the lowest RNA and protein expression for these genes. For further in vitro experiments, we selected the OPM-2 and RPMI-8226 cells as these cell lines showed differential PYCR expression. To evaluate whether hypoxia, a key component of metabolic drug resistance, increased proline metabolism, we performed a tracer study in which we supplemented RPMI-8226 cells with ^13^C-labeled glutamine. During cell growth, ^13^C-glutamine is transformed into several metabolites such as glutamate, arginine, and proline. The relative amount of ^13^C found in these metabolites correlates with their intracellular flux. In this tracer study, we observed an increased glutamine-to-proline conversion stimulated by hypoxia (Fig. 2C). Proline content, measured in OPM-2 and RPMI-8226 cells by ELISA, was also increased after 48 h of hypoxic culture (Fig. 2D). Therefore, all further in vitro experiments were performed in 1% oxygen, as this closely resembles the hypoxic nature of the BM.

### PYCR1 inhibition reduces MM cell proliferation in vitro

**Fig. 3:**
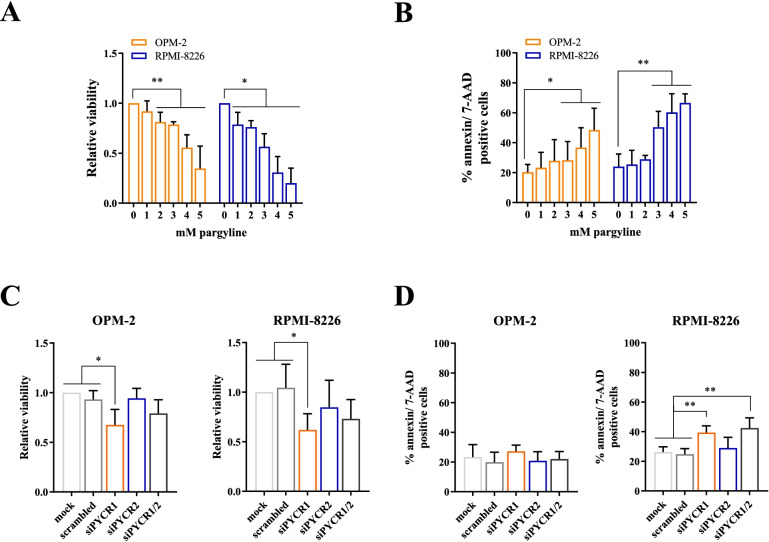
PYCR1 inhibition by pargyline and siPYCR1 reduces MM cell viability in vitro. OPM-2 and RPMI-8226 were cultured in a hypoxic environment during 48 h (pargyline) and 72 h (siRNA). SiRNA-mediated knockdown was established using 20 nM siPYCR1 and/or 50 nM siPYCR2. **A** Viability was measured by CellTiter Glo assay after cells were treated with increasing doses of pargyline (*n* = 3). **B** Pargyline-induced apoptosis was measured using flow cytometry by staining for Annexin V and 7-AAD (*n* = 3). **C** Viability was measured by CellTiter Glo assay after treatment with siRNA for PYCR1 and/or PYCR2 (*n* = 5). **D** Apoptosis was measured using flow cytometry by staining for Annexin V and 7-AAD after treatment with siRNA for PYCR1 and/or PYCR2 (*n* = 5). All experiments were performed in hypoxic conditions. Significance was determined by Mann–Whitney U test and one-way ANOVA. * *p* ≤ 0.05, ** *p* ≤ 0.01

**Fig. 4:**
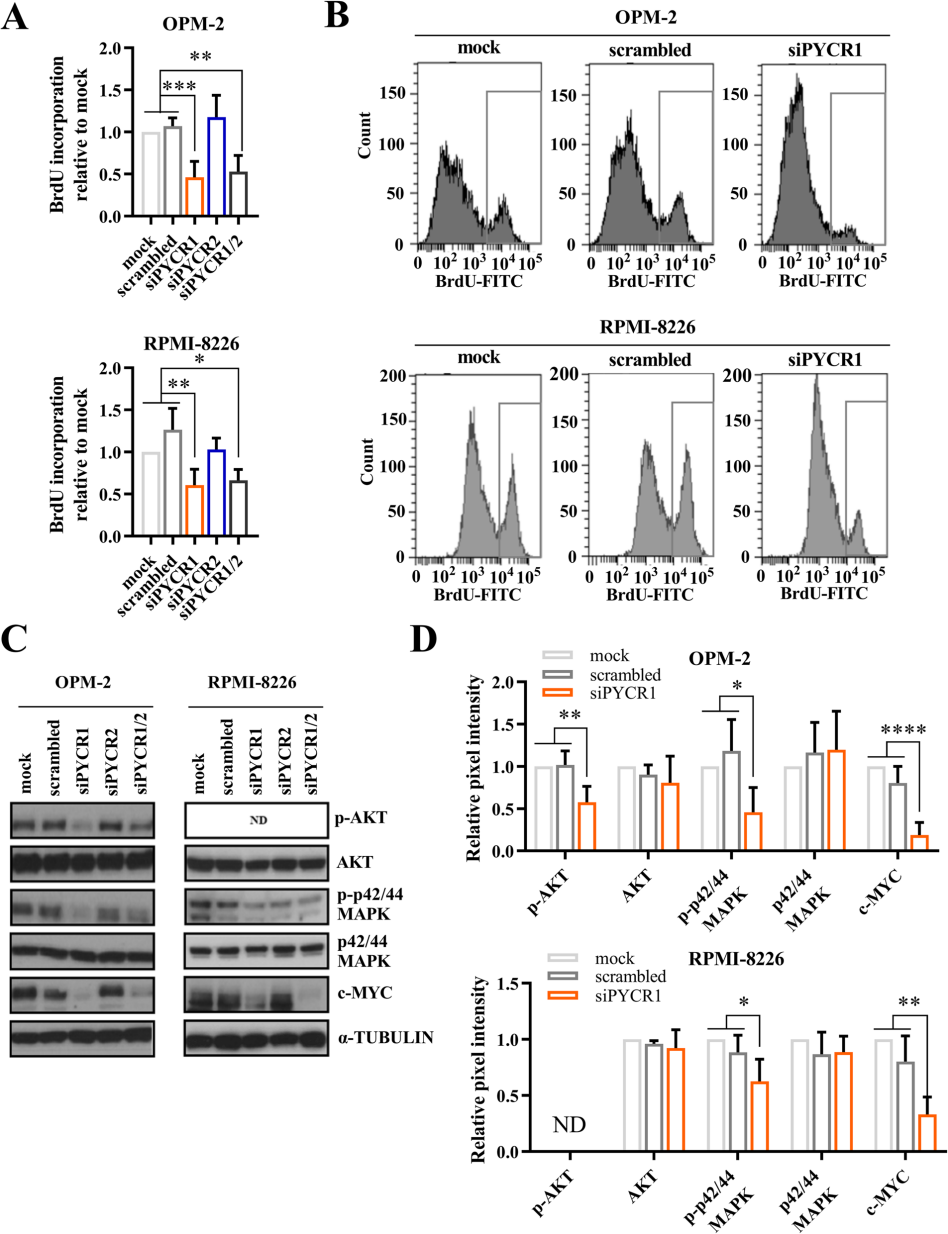
PYCR1 interference reduces proliferation in vitro. OPM-2 and RPMI-8226 were cultured in a hypoxic environment and knockdown was established using 20 nM siPYCR1 and/or 50 nM siPYCR2. **A**, **B** Relative BrdU incorporation was measured by BrdU-FITC positivity on flow cytometry after 72 h (OPM-2) and 48 h of culture (RPMI-8226) (*n* = 5). **C)** Survival and proliferation markers were measured on protein level by western blotting after 72 h of culture (*n* = 5). **D** Bar graphs represent the pixel intensity measured by Image Studio Lite v5.2. All graphs are shown relative to mock (*n* = 5). All experiments were performed in hypoxic conditions. ND = not detected. Significance was determined by one-way ANOVA. * *p* ≤ 0.05, ** *p* ≤ 0.01, *** *p* ≤ 0.001, **** *p* ≤ 0.0001

To investigate whether PYCR1 inhibition influences MM viability, we first treated OPM-2 and RPMI-8226 cells with increasing doses of the clinically relevant compound pargyline, a PYCR1 small molecule inhibitor [32]. Pargyline is an irreversible selective monoamine oxidase B inhibitor and used to treat hypertension. It was first identified as a PYCR1 inhibitor in 2019 through screening on the breast cancer cell line SUM-159-PT [32]. However, its’ exact working mechanism has not yet been identified. When treating OPM-2 and RPMI-8226 with pargyline, we observed a dose-dependent decrease in viability and increase in apoptosis (Fig. 3A-B) after 48 h of hypoxia. We confirmed that pargyline can reduce proline production (Suppl Fig. 1). Next, we examined whether silencing the different PYCR enzymes, using specific siRNAs, had the same effect. We silenced either PYCR1, PYCR2 or PYCR1/2 simultaneously and cultured the cells for 72 h in a hypoxic environment (Suppl Fig. 2A-B). Remarkably, knockdown of PYCR1 also decreased PYCR2 expression on both RNA and protein level. SiRNA-mediated knockdown of PYCR1 significantly reduced MM cell viability in both cell lines after 72 h of hypoxic culture (Fig. 3C). PYCR1 interference increased apoptotic cell death in the RPMI-8226 cells, but not in OPM-2 (Fig. 3D). BrdU incorporation was also significantly decreased when PYCR1 expression was silenced, confirming a decrease in proliferation (Fig. 4A-B). On protein level, several proliferation and survival pathways were altered as we observed a decrease in p-AKT, p-p42/44 MAPK and c-MYC levels (Fig. 4C-D). For PYCR2 however, knockdown did not significantly alter viability, apoptosis and proliferation and only small decreases in proliferation and survival pathways were observed (Figs. 3C-D and Fig. 4A). In case of simultaneous PYCR1/2 knockdown, viability was not altered, and siPYCR1/2 only significantly increased apoptosis in RPMI-8226 (Fig. 3D). A decrease in BrdU incorporation was also observed in both cell lines, although compared to PYCR1 knockdown, no added effect was seen (Fig. 4A). Similarly, PYCR1/2 knockdown did not increase the effects on p-AKT, p-p42/44 MAPK and c-MYC, therefore we continued with only silencing PYCR1 to examine downstream pathways (Fig. 4C).

### Knockdown of PYCR1 inhibits PRAS40-mediated protein synthesis

To identify the pathway of action through which PYCR1 inhibits myeloma growth, we performed a proteome profiling regarding phospho-kinases on OPM-2 cells. Similar to previous experiments, OPM-2 cells were treated with siPYCR1 and cultured for 72 h in a hypoxic environment. The proteome profiling confirmed previously observed results on the p-p42/44 MAPK pathway (Suppl Fig. 3). We also noticed > 50% decrease in p-STAT1, p-p70 and p-PRAS40. Additionally, several other reductions in phospho-kinases were observed. Src kinases phospho-lck, p-lyn and chromatine modifier p-msk1/2 showed a ~ 50% decrease when PYCR1 was silenced. Phospho-yes, p-p53, p-PYK2 and p-STAT6 were also downregulated.

**Fig. 5:**
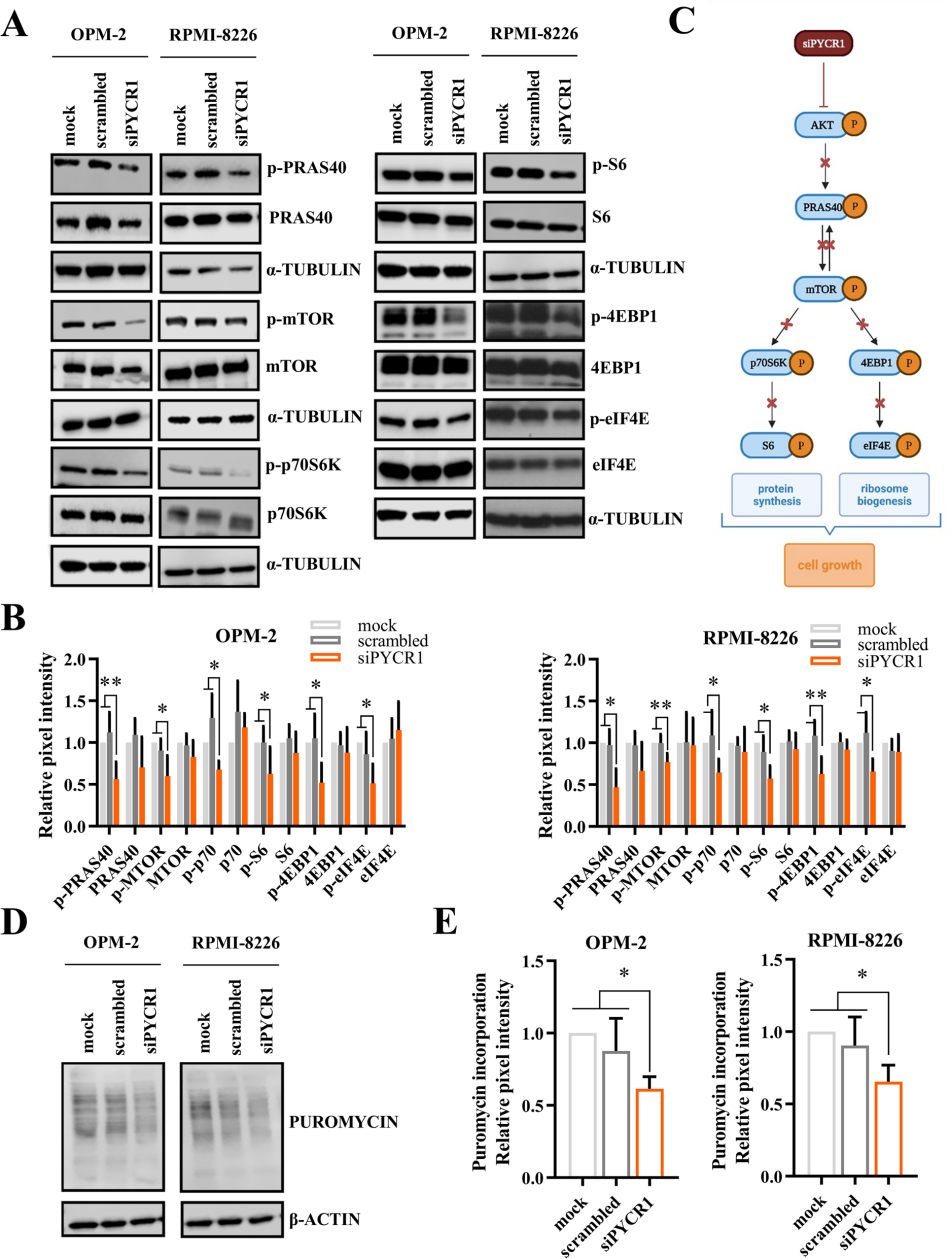
PYCR1 interference inhibits protein synthesis by downregulating the PRAS40 pathway. OPM-2 and RPMI-8226 were incubated with 20 nM siPYCR1 and cultured in a hypoxic environment. **A**, **B** Western blot analysis of protein synthesis-related proteins, isolated after 72 h (or 96 h in case of OPM-2 p-eIF4E, eIF4E, p-4EBP1, 4EBP1) of culture. Bar graphs represent the pixel intensity measured by Image Studio Lite v5.2. All graphs are shown relative to mock (*n* ≤ 5). **C** Overview of the PRAS40 pathway. Created with BioRender.com. **D**, **E** Western blot analysis of puromycin uptake 72 h after PYCR1 knockdown is initiated (*n* = 5). All experiments were performed in hypoxic conditions. Significance was determined by Kruskal–Wallis or one-way ANOVA * *p* ≤ 0.05, ** *p* ≤ 0.01

Changes in p-p70 and p-PRAS40 expression were confirmed by western blot for OPM-2 and RPMI-8226. We further explored the PRAS40 pathway and found a decrease in several downstream targets of PRAS40: p-S6, p-4EBP1 and p-eIF4E (Fig. 5A-C), suggesting the involvement of PYCR1 and proline production in protein synthesis. We used the non-radioactive SUnSET method to validate this effect on protein synthesis [33]. Hereby, we supplemented the cells with puromycin during 30 min, followed by cell isolation, lysis and western blot. The amount of puromycin uptake is directly correlated with protein synthesis. We observed a significant decrease in puromycin uptake when PYCR1 was silenced (Fig. 5D-E), as well as when MM cells were treated with the PYCR1 inhibitor pargyline (Suppl Fig. 4A-B).

### PYCR1 inhibition increases bortezomib-mediated apoptotic cell death in vitro and in vivo

**Fig. 6:**
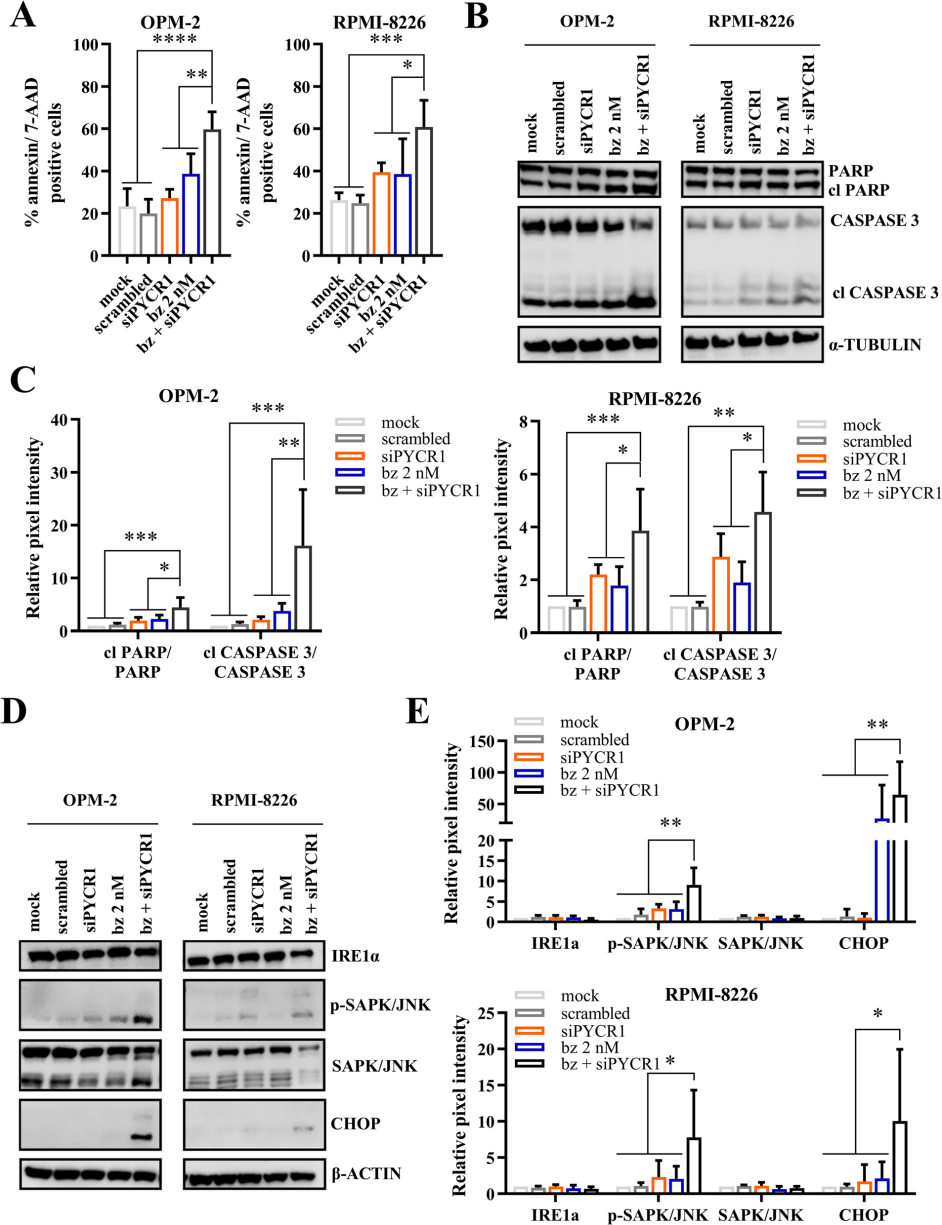
PYCR1 interference increases bortezomib-mediated cell death in vitro. **A** OPM-2 and RPMI-8226 were cultured in a hypoxic environment for 72 h and treated with siRNA for PYCR1 and bortezomib. Apoptosis was measured using flow cytometry by staining for Annexin V and 7-AAD (*n* = 5). **B-C** OPM-2 and RPMI-8226 were cultured in a hypoxic environment for 72 h and treated with siRNA for PYCR1 and bortezomib. Apoptosis markers PARP and CASPASE 3 were measured on protein level by western blotting. Bar graphs represent the pixel intensity measured by Image Studio Lite v5.2. All graphs are shown relative to mock (*n* = 5). **D-E** OPM-2 and RPMI-8226 were cultured in a hypoxic environment for 72 h and treated with siRNA for PYCR1 and bortezomib. UPR markers IRE1a, p-SAPK/JNK and CHOP were measured on protein level by western blotting. Bar graphs represent the pixel intensity measured by Image Studio Lite v5.2. All graphs are shown relative to mock (*n* ≤ 6). All experiments were performed in hypoxic conditions. bz = bortezomib. Significance was determined by one-way ANOVA. * *p* ≤ 0.05, ** *p* ≤ 0.01, *** *p* ≤ 0.001, **** *p* ≤ 0.0001

**Fig. 7:**
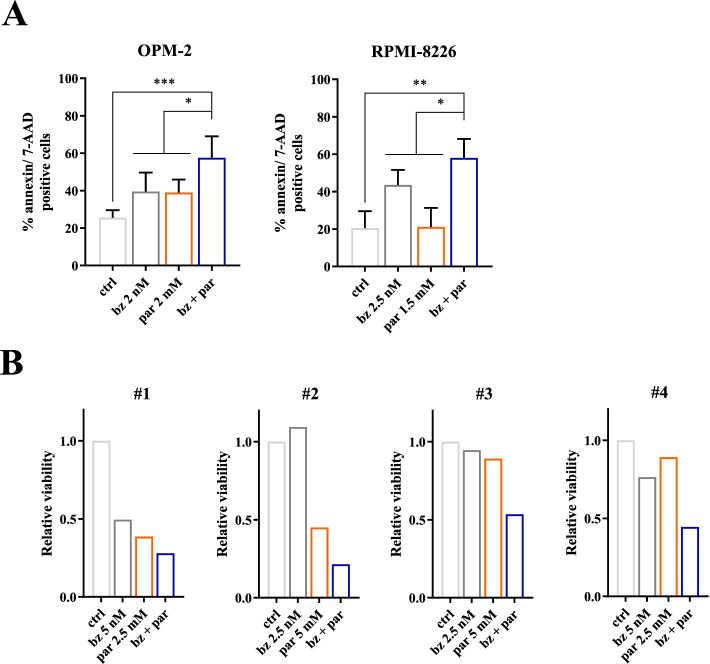
PYCR1 inhibition increases bortezomib-mediated cell death in vitro and ex vivo. **A** OPM-2 and RPMI-8226 were cultured for 48 h in a hypoxic environment and treated with bortezomib and pargyline. Apoptosis was measured using flow cytometry by staining for Annexin V and 7-AAD (*n* = 5). **B** CD138^+^ MM cells from several patient samples were cultured for 24 h and treated with bortezomib and pargyline. Viability was measured by CellTiter Glo assay (*n* = 4). All experiments were performed in hypoxic conditions. ctrl = control, bz = bortezomib, par = pargyline. Significance was determined by one-way ANOVA. * *p* ≤ 0.05, ** *p* ≤ 0.01, *** *p* ≤ 0.001

To investigate whether PYCR1 inhibition could be added as part of a combination strategy in a clinical setting, we combined PYCR1 inhibition with one of the current standard-of-care agents bortezomib, a proteasome inhibitor. PYCR1 knockdown significantly enhanced the bortezomib-mediated effects on apoptosis (Fig. 6A, Suppl Fig. 5). PYCR1/2 knockdown showed similar effects on apoptosis (Suppl Fig. 6), while we observed no difference when PYCR2 was silenced (Suppl Fig. 6). On protein level, we saw a significant increase in cleaved PARP/total PARP and cleaved Caspase 3/total Caspase 3 levels when PYCR1 silencing was combined with bortezomib (Fig. 6B-C). We also investigated whether the combination therapy affected the PRAS40 pathway. Here, we found a non-significant decrease in p-PRAS40 and p-4EBP1 comparing the combination therapy of bz + siPYCR1 to both single agents (Suppl Fig. 7A-B). The reduction in protein synthesis, measured by puromycin uptake, was only significant compared to mock and scrambled samples (Suppl Fig. 7C-D). As these decreases in the PRAS40 pathway were not significant, we looked at other possible synergistic pathways. Bortezomib is a proteasome inhibitor and has been shown to trigger the unfolded protein response (UPR) pathway, causing cell death in cancer cells [34, 35]. This pathway plays an important role in protein synthesis by influencing the modification, folding and assembly of proteins [36]. When investigating this pathway, we found that bz + siPYCR1 significantly induced the UPR pathway by increasing CHOP and p-SAPK/JNK on protein level (Fig. 6D-E). Both increases were significant compared to all single agents, leading to apoptosis. Next, we verified whether the PYCR1 inhibitor pargyline also increased bortezomib-mediated cell death. We indeed observed a significant increase in apoptotic cells when MM cells were treated with both bortezomib and pargyline (Fig. 7A, Suppl Fig. 8). Moreover, this combination therapy was also effective on primary MM samples (CD138^+^). While every patient sample responded differently to the treatments, the combination did further decrease the viability of the MM cells (Fig. 7B). Patient and disease characteristics can be found in Suppl table 2.

**Fig. 8:**
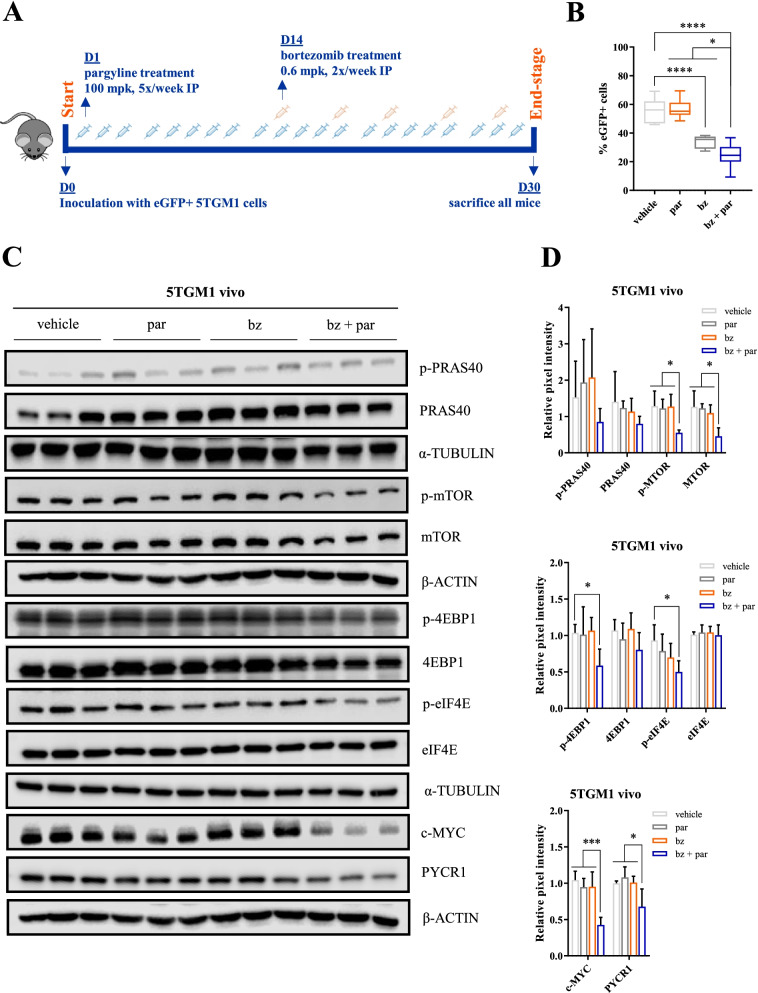
PYCR1 inhibition by pargyline enhances bortezomib sensitivity in vivo. **A** Treatment overview of 5TGM1 mice (*n* = 9/group). 5TGM1 mice were treated 5x/week with pargyline (100 mpk) and/or bortezomib 2x/week (0.6 mpk, starting 14 days after inoculation). All mice were sacrificed at day 30. **B)** Tumor burden was measured based upon eGFP positivity by flow cytometry (n = 9). eGFP positivity reflects the percentage of MM cells (= 5TGM1 vivo) present in the BM microenvironment. **C-D** Western blot analysis of protein synthesis-related proteins, originating from 5TGM1 vivo cells. Bar graphs represent the pixel intensity measured by Image Studio Lite v5.2. All graphs are shown relative to vehicle (*n* = 3 shown, *n* = 5 total). par = pargyline, bz = bortezomib, mpk = milligram per kilogram. Significance was determined by one-way ANOVA. * *p* ≤ 0.05, *** *p* ≤ 0.001 **** *p* ≤ 0.0001

We finally evaluated whether these observed in vitro effects could also be translated to an in vivo setting. We first treated the murine 5TGM1 cell line in vitro with pargyline, and similar to the human MM cell lines, we observed an increase in apoptosis when pargyline treatment was combined with bortezomib (Suppl Fig. 9A-B). We then treated the syngeneic immunocompetent 5TGM1 bearing mice with bortezomib and/or pargyline and evaluated effects on tumor burden when vehicle mice reached end-stage, 30 days post-inoculation (Fig. 8A). We evaluated tumor load by analysing eGFP positivity on flow cytometry. Although pargyline as a single agent did not alter tumor load (Fig. 8B), we did observe a significant decrease in tumor load for the combination therapy compared to both single agents. On protein level, combination therapy significantly reduced p-4EBP1 and p-eIF4E levels, as well as p-mTOR, c-MYC and PYCR1 levels (Fig. 8C-D, Suppl Fig. 9C-D).

## Discussion

In this study, we first explored whether PYCR expression contributes to MM cell survival. MM cells reside in the BM, which is hypoxic in nature. Therefore, MM cells have an altered metabolism, related to hypoxia. In 2019, Junker et al. demonstrated that advanced MM progression was associated with metabolic changes, linked to higher levels of hypoxia. They showed a high increase of glutamine uptake in MM cells from patients with high BM infiltration (> 40%, MM_high_). Moreover, MM_high_ cells contain almost triple the amount of proline levels compared to B cells. Furthermore, PYCR1 and PYCR3 expression were upregulated in MM cells with high BM infiltration compared to low BM infiltration (< 20%), while PYCR2 expression was found to be higher in MM compared to B cells [37]. We confirmed these findings with our tracer study in RPMI-8226 that showed an increased glutamine-to-proline conversion after 48 h of hypoxic culture, which indicates an increased activity of the PYCR enzymes. All further in vitro experiments were performed in 1% 0_2_, to closely resemble the hypoxic nature of the BM.

We assessed the MMRF CoMMpass trial whether PYCR1 and PYCR2 mRNA are associated with adverse survival in MM patients. The PYCR enzymes have been previously associated with cancer progression. In breast cancer, PYCR1 has been correlated with poor prognosis, and this for both ER + and ER- breast cancers [27]. Moreover, microarray data from the GeneChip Oncology Database [38] has shown that PYCR1 is one of the most frequently over-expressed metabolic genes in human tumors [27]. Overexpression of PYCR1 has also been shown in bladder cancer [39], gastric cancer [40], NSCLC [41] as well as hepatocellular cancer [28].

We investigated whether relapsed/refractory patients express different levels of PYCR1/2 mRNA compared to newly diagnosed patients. Although small, PYCR1 showed significantly higher levels when patients relapse.

To further validate the clinical potential of PYCR targeting, we investigated PYCR protein expression in the CD138^+^ fraction of primary patient samples. Here we observed PYCR1 expression in every sample that we tested. However, PYCR2 expression was detected in only two out of five tested patients.

To investigate the role of PYCR1/2 in MM cell biology, we performed knockdown experiments where we either silenced PYCR1, PYCR2 or PYCR1/2 simultaneously and evaluated effects on apoptosis and proliferation. We chose OPM-2 and RPMI-8226 cells as they belong to different MM subclasses and show a different PYCR1/2 expression. As a single agent, the knockdowns did not alter apoptosis in OPM-2, but siPYCR1 did increase apoptosis in RPMI-8226, confirming previously seen effects on apoptosis in prostate cancer [42], hepatocellular cancer [28] and melanoma [43]. Knockdown of PYCR1 also increased bortezomib efficacy in both cell lines. PYCR1 interference reduced several proliferation and survival markers like p-AKT, p-p42/44 MAPK and c-MYC, which coincided with a significant decrease in BrdU incorporation. In literature, PYCR1 interference has been shown to decrease p-AKT levels in bladder cancer cell lines [39], papillary renal cell carcinoma [44] and in melanoma [43], while it also reduces cell growth in hepatocellular cancer cell lines [28]. PYCR2 silencing did not significantly alter apoptosis nor proliferation, indicating that it is not involved in MM progression. Therefore, we continued by only investigating the downstream signaling induced by PYCR1 silencing.

To obtain a better knowledge of the underlying pathway responsible for the observed effects on proliferation and apoptosis, we performed a kinase proteome profiling, comparing OPM-2 scrambled cells to siPYCR1 transfected cells. We observed decreases in p-p70 S6 kinase and p-PRAS40, which are both involved in protein synthesis. We further investigated this pathway, and found a decrease in p-PRAS40, p-mTOR, p-p70, p-S6, p-4EBP1 and p-eIF4E by western blot, suggesting a dysregulation of the protein synthesis pathway. This coincided with a reduction in puromycin uptake. Previously, PYCR1 knockdown has been shown to reduce p-mTOR in renal cell carcinoma [44] and p70 in melanoma [45]. However, we are the first to show in any cancer that PYCR1 interference causes a systematic decrease of the PRAS40 pathway, effectively inhibiting cell growth and mRNA translation. The PI3K/AKT/mTOR pathway is well established in MM as an important mediator of drug resistance and MM pathogenesis [46, 47]. Inhibition of p-p70, p-S6, p-4EBP1 and p-eIF4E has been show to impair MM progression and induce cell death [46, 48]. For instance, our research group has previously shown that G9a/GLP targeting in myeloma inhibits p-mTOR, followed by a decrease in p-4EBP1 and p-eIF4E, leading to MM apoptosis [49]. However, the PRAS40-mediated protein synthesis pathway as a whole has not been studied yet in MM. Other pathways shown to be altered by PYCR1 interference are the p38 MAPK pathway in NSCLC [41] and the IRS1/JNK pathway in hepatocellular cancer [28], although we could not confirm this in MM (data not shown).

The link between PYCR, proline metabolism and the oncogenic transcription factor c-MYC has been studied in other cancers. c-MYC increases cancer cell proliferation by upregulating glutaminase, an enzyme involved in the conversion of glutamine to glutamate. Moreover, Liu et al. [43] showed in lymphoma that MYC is able to increase expression of PYCR1-3 and pyrroline-5-carboxylate synthase (P5CS), stimulating the glutamine-to-proline biosynthesis. Silencing of MYC reduced expression of all four enzymes on both mRNA and protein level, leading to reduced proline biosynthesis, followed by reduced glycolysis and ATP production [43, 50, 51]. Here, we showed that siPYCR1 is also able to reduce c-MYC expression, suggesting a possible feedback loop.

MYC expression is regulated through the PI3K-mTOR pathway [52, 53]. It has been shown that mTOR stabilizes MYC expression and causes MYC translation. c-MYC possesses a short half-life of around 15–30 min and is strongly influenced by compounds affecting protein synthesis. In order to keep up its highly expressed presence, c-MYC needs to be continuously synthesized, which is dependent on mTOR-mediated phosphorylation of 4EBP1 [52, 53]. Combined with our data, siPYCR1 can reduce c-MYC expression through inhibition of PI3K/mTOR and reduced 4EBP1 phosphorylation.

Combination experiments with siPYCR1 and bortezomib further increased apoptosis and demonstrated a non-significant further reduction in both the PRAS40 pathway and puromycin uptake. More importantly, the combination strategy increased UPR components p-SAPK/JNK and CHOP, causing ER stress and programmed cell death. Previously, bortezomib as a proteasome inhibitor, has been shown to trigger the UPR response by accumulation of misfolded proteins, inducing cell death [34, 35].

To demonstrate clinical relevance, we performed experiments using the small molecule inhibitor pargyline to block PYCR1. In our study, pargyline was able to reduce proline production and showed a dose-dependent increase in apoptosis and puromycin uptake, and increased bortezomib efficacy in vitro, similar to our knockdown experiments. In vivo, pargyline reduced the tumor load when combined with bortezomib. Pathway investigation showed a significant decrease of p-4EBP1 and p-eIF4E, the final components of PRAS40 pathway. We also explored the expression of the other pathway components. Here we mostly found decreases in p-PRAS40, p-mTOR, p-p70 and p-S6, although only significant for p-mTOR. This might be due to the later timepoint of analysis. Additionally, c-MYC and PYCR1 levels were also significantly decreased.

As pargyline is an anti-hypertension agent and has also been used as a lysine-specific histone demethylase 1 inhibitor [54, 55], we cannot exclude off-target effects. However, we were able to prove that pargyline reduces intracellular proline concentration at doses that causes only limited apoptosis. Moreover, RPMI-8226 cells are more sensitive to pargyline treatment than OPM-2 cells, which is in concordance to the knockdown experiments where siPYCR1 already caused apoptosis as a single agent in RPMI-8226, but not in OPM-2. Currently, more specific PYCR1 inhibitors are being identified and manufactured [32, 56].

## Conclusions

In summary, we demonstrate a role for PYCR1 in MM cell growth and survival. PYCR1 is an adverse prognostic factor in MM showing higher expression in relapsed patients. The PYCR1 inhibitor pargyline shows single agent activity in HMCL. PYCR1 interference reduced myeloma cell proliferation and survival by inhibiting PRAS40-mediated protein synthesis. PYCR1 inhibition increased bortezomib efficacy, both in vitro and in vivo*,* by triggering an UPR response. Our findings suggest PYCR1 as new therapeutic target in MM treatment.

## Supplementary Information

**Additional file 1: Supplemental Figure 1. **Pargyline reduces proline production. **Supplemental Figure 2. **SiRNA-mediated knockdown reduces PYCR1 and PYCR2 expression on mRNA and protein level.** Supplemental Figure 3. **PYCR1 interference inhibits protein synthesis by downregulating the PRAS40 pathway. **Supplemental Figure 4. **PYCR1 inhibition by pargyline inhibits protein synthesis. **Supplemental Figure 5. **PYCR1 inhibition increases bortezomib-mediated apoptosis in MM cell lines. **Supplemental Figure 6. **PYCR1 interference, but not PYCR2, increases bortezomib-mediated apoptosis in MM cell lines. **Supplemental Figure 7. **Combination therapy of siPYCR1 and bortezomib non-significantly decreases protein synthesis. **Supplemental Figure 8. **PYCR1 inhibition by pargyline increases bortezomib-mediated apoptosis in MM cell lines. **Supplemental Figure 9. **PYCR1 inhibition by pargyline increases apoptosis in 5TGM1 *in vitro *and *in vivo*. **Supplementary Table 1. **Patient and disease characteristics for MM patients included in the protein PYCR expression investigation. **Supplementary Table 2. **Patient and disease characteristics for MM patients which bone marrow aspirates were used to test pargyline effects on viability.

## Data Availability

Source data are provided with this paper. All other data supporting the findings of the study are available from the corresponding authors upon request.

## Abbreviations

MM: Multiple myeloma
PYCR: Pyrroline-5-carboxylate reductase
BM: Bone marrow
LDHA: Lactate dehydrogenase A
HIF1α: Hypoxia-inducible factor alpha
KDM3A: Histone lysine demethylase 3A
NSCLC: Non-small cell lung cancer
ER: Estrogen receptor
HMCL: Human myeloma cell line
MEC: Metabolomics Expertise Centrum
BrdU: Bromodeoxyuridine
UPR: Unfolded protein response
