# Supplementary material for: Pyrroline-5-Carboxylate Reductase 1: a novel target for sensitizing multiple myeloma cells to bortezomib by inhibition of PRAS40-mediated protein synthesis
Source: J Exp Clin Cancer Res. 2022 Feb 1;41:45. doi: 10.1186/s13046-022-02250-3 (PMC8805317; doi:10.1186/s13046-022-02250-3)
Supplement: Supplementary file 1 — Additional file 1: Supplemental Figure 1. Pargyline reduces proline production. Supplemental Figure 2. SiRNA-mediated knockdown reduces PYCR1 and PYCR2 expression on mRNA and protein level. Supplemental Figure 3. PYCR1 interference inhibits protein synthesis by downregulating the PRAS40 pathway. Supplemental Figure 4. PYCR1 inhibition by pargyline inhibits protein synthesis. Supplemental Figure 5. PYCR1 inhibition increases bortezomib-mediated apoptosis in MM cell lines. Supplemental Figure 6. PYCR1 interference, but not PYCR2, increases bortezomib-mediated apoptosis in MM cell lines. Supplemental Figure 7. Combination therapy of siPYCR1 and bortezomib non-significantly decreases protein synthesis. Supplemental Figure 8. PYCR1 inhibition by pargyline increases bortezomib-mediated apoptosis in MM cell lines. Supplemental Figure 9. PYCR1 inhibition by pargyline increases apoptosis in 5TGM1 in vitro and in vivo. Supplementary Table 1. Patient and disease characteristics for MM patients included in the protein PYCR expression investigation. Supplementary Table 2. Patient and disease characteristics for MM patients which bone marrow aspirates were used to test pargyline effects on viability. [file 13046_2022_2250_MOESM1_ESM.pptx]

## Slide 1
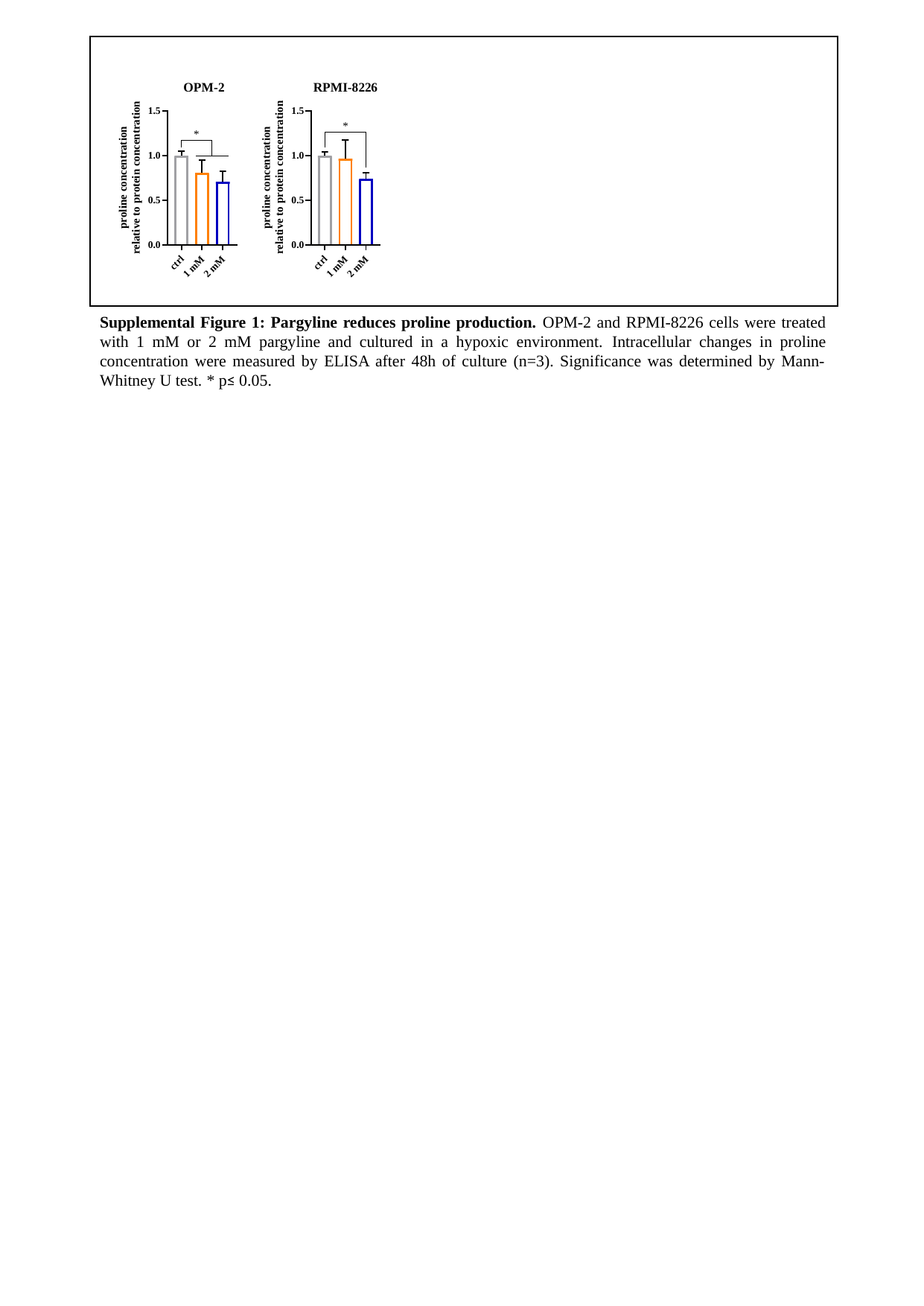

Supplemental Figure 1: Pargyline reduces proline production. OPM-2 and RPMI-8226 cells were treated with 1 mM or 2 mM pargyline and cultured in a hypoxic environment. Intracellular changes in proline concentration were measured by ELISA after 48h of culture (n=3). Significance was determined by Mann-Whitney U test. * p≤ 0.05.

## Slide 2
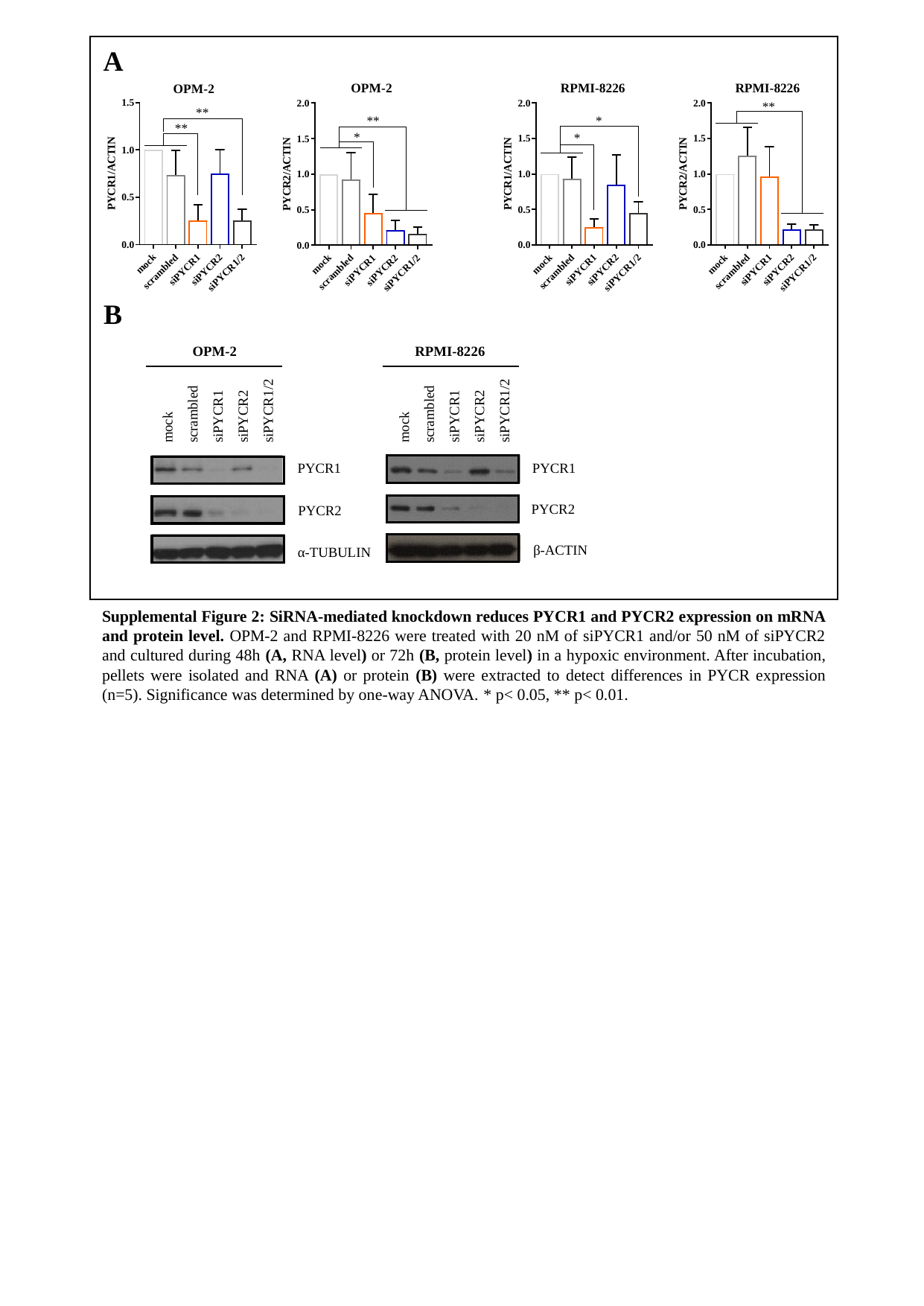

A
B
RPMI-8226
OPM-2
mock
scrambled
siPYCR1
siPYCR2
siPYCR1/2
mock
scrambled
siPYCR1
siPYCR2
siPYCR1/2
PYCR1
PYCR1
PYCR2
PYCR2
β-ACTIN
α-TUBULIN
Supplemental Figure 2: SiRNA-mediated knockdown reduces PYCR1 and PYCR2 expression on mRNA and protein level. OPM-2 and RPMI-8226 were treated with 20 nM of siPYCR1 and/or 50 nM of siPYCR2 and cultured during 48h (A, RNA level) or 72h (B, protein level) in a hypoxic environment. After incubation, pellets were isolated and RNA (A) or protein (B) were extracted to detect differences in PYCR expression (n=5). Significance was determined by one-way ANOVA. * p< 0.05, ** p< 0.01.

## Slide 3
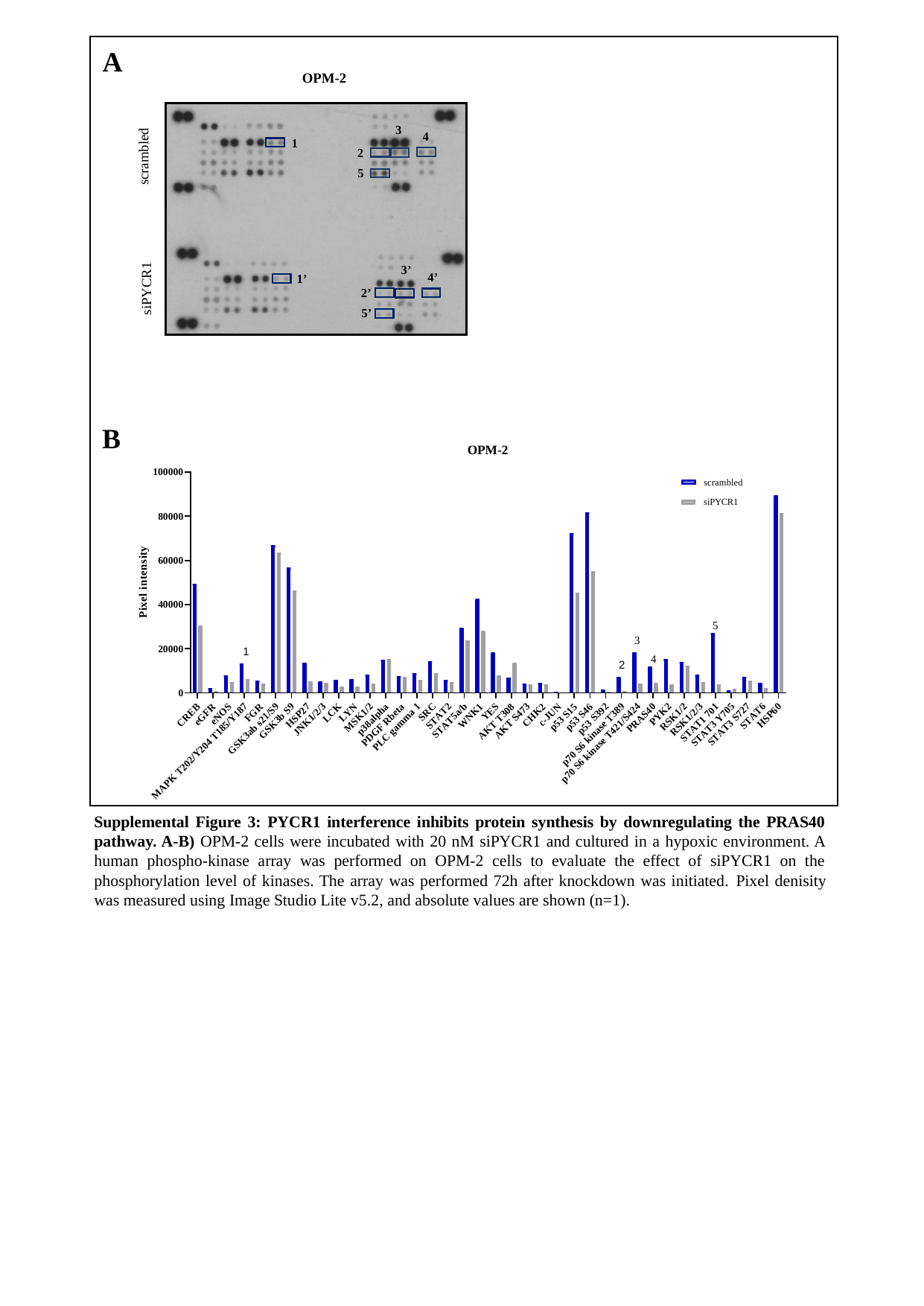

A
OPM-2
3
4
1
2
scrambled
5
3’
4’
1’
siPYCR1
2’
5’
B
Supplemental Figure 3: PYCR1 interference inhibits protein synthesis by downregulating the PRAS40 pathway. A-B) OPM-2 cells were incubated with 20 nM siPYCR1 and cultured in a hypoxic environment. A human phospho-kinase array was performed on OPM-2 cells to evaluate the effect of siPYCR1 on the phosphorylation level of kinases. The array was performed 72h after knockdown was initiated. Pixel denisity was measured using Image Studio Lite v5.2, and absolute values are shown (n=1).

## Slide 4
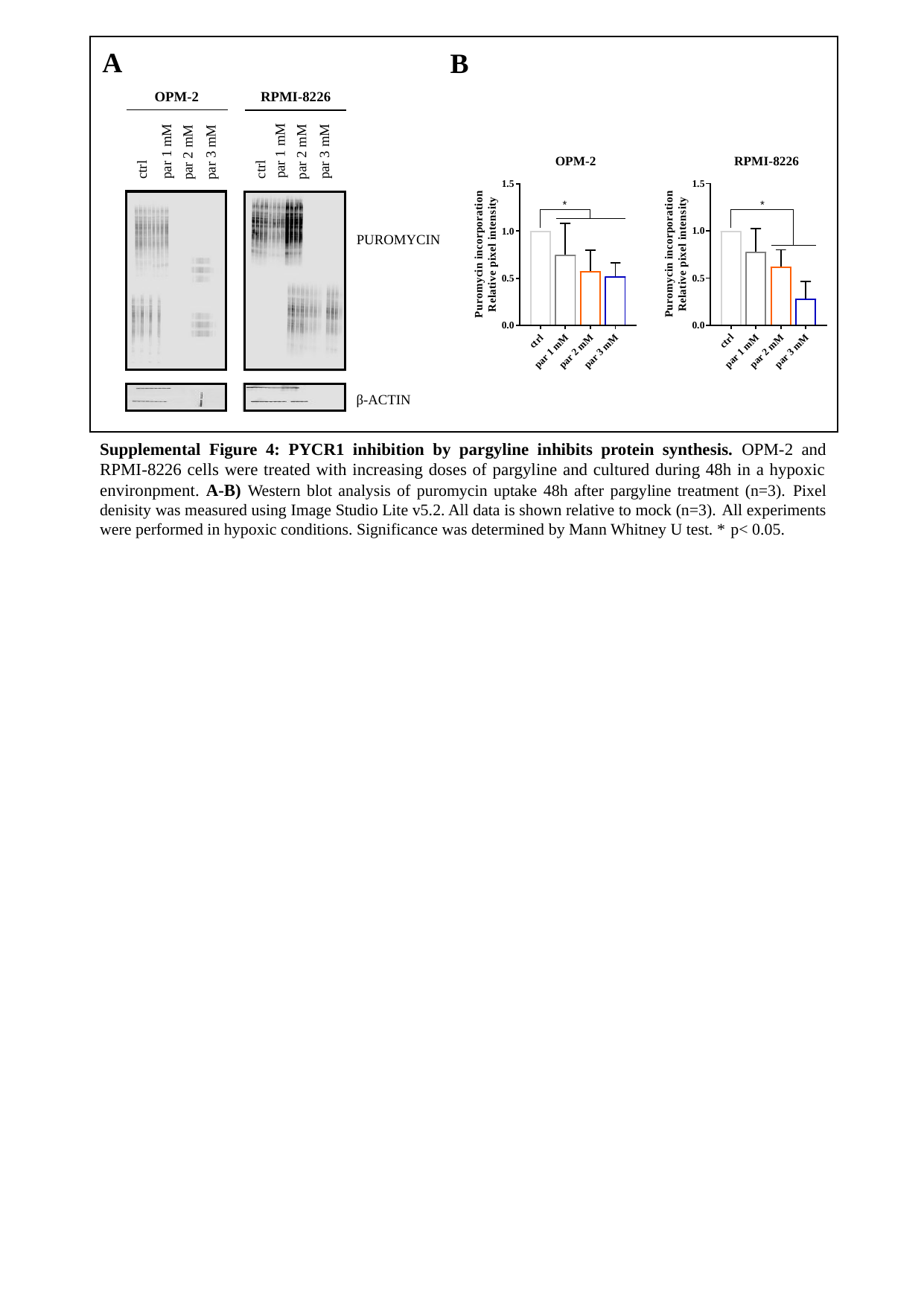

A
B
OPM-2
RPMI-8226
par 1 mM
par 1 mM
par 3 mM
par 2 mM
par 3 mM
par 2 mM
ctrl
ctrl
PUROMYCIN
β-ACTIN
Supplemental Figure 4: PYCR1 inhibition by pargyline inhibits protein synthesis. OPM-2 and RPMI-8226 cells were treated with increasing doses of pargyline and cultured during 48h in a hypoxic environpment. A-B) Western blot analysis of puromycin uptake 48h after pargyline treatment (n=3). Pixel denisity was measured using Image Studio Lite v5.2. All data is shown relative to mock (n=3). All experiments were performed in hypoxic conditions. Significance was determined by Mann Whitney U test. * p< 0.05.

## Slide 5
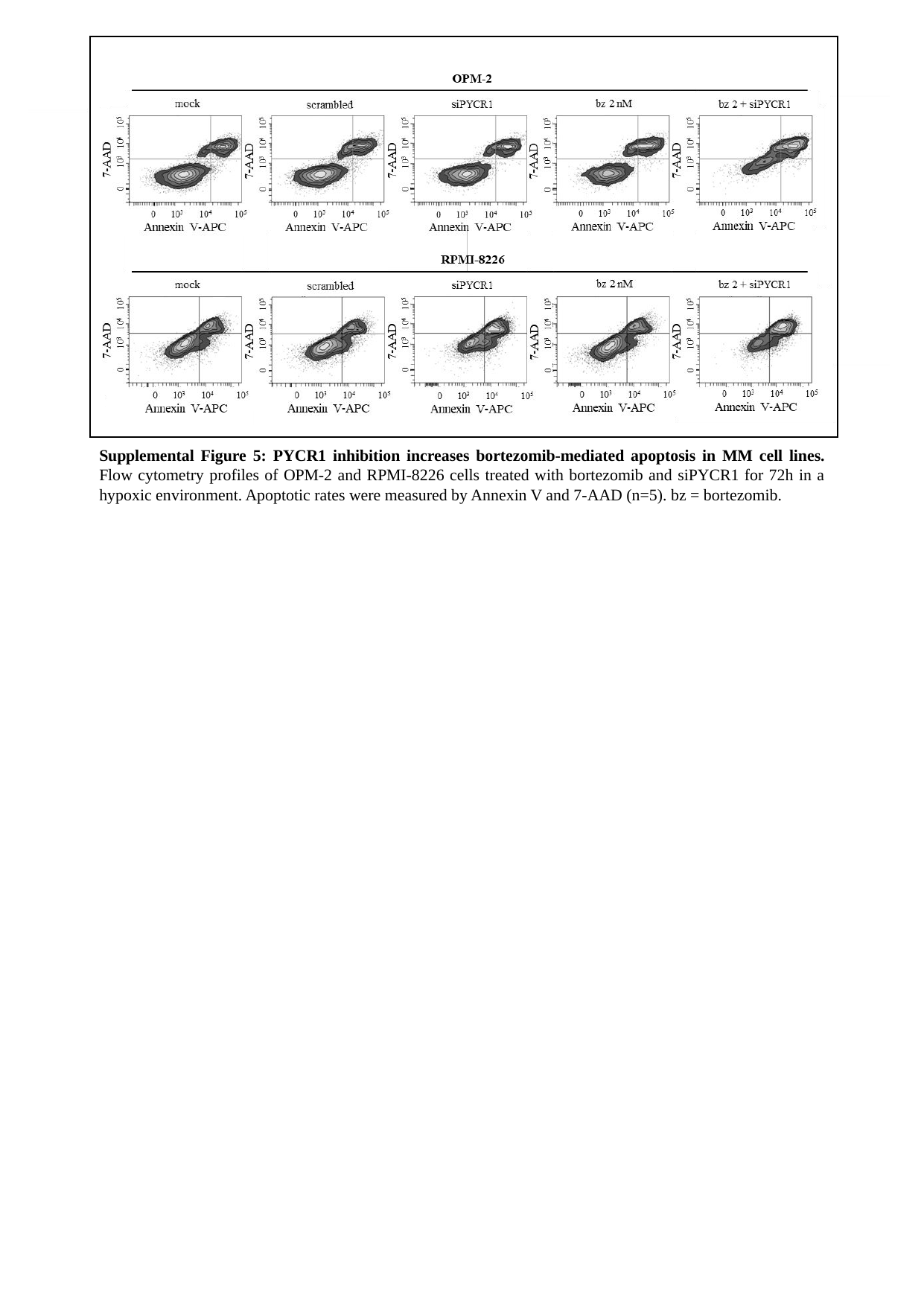

Supplemental Figure 5: PYCR1 inhibition increases bortezomib-mediated apoptosis in MM cell lines. Flow cytometry profiles of OPM-2 and RPMI-8226 cells treated with bortezomib and siPYCR1 for 72h in a hypoxic environment. Apoptotic rates were measured by Annexin V and 7-AAD (n=5). bz = bortezomib.

## Slide 6
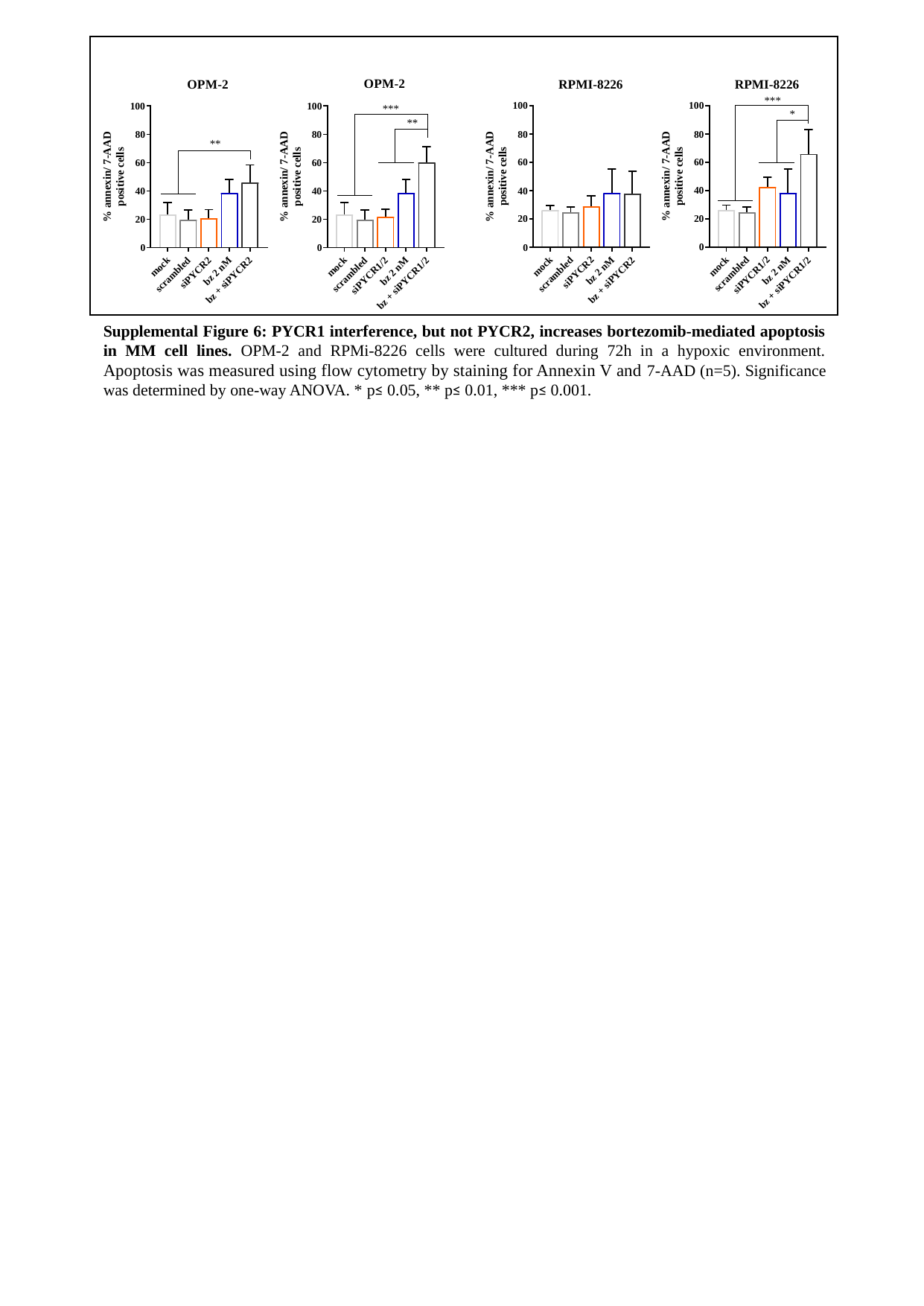

Supplemental Figure 6: PYCR1 interference, but not PYCR2, increases bortezomib-mediated apoptosis in MM cell lines. OPM-2 and RPMi-8226 cells were cultured during 72h in a hypoxic environment. Apoptosis was measured using flow cytometry by staining for Annexin V and 7-AAD (n=5). Significance was determined by one-way ANOVA. * p≤ 0.05, ** p≤ 0.01, *** p≤ 0.001.

## Slide 7
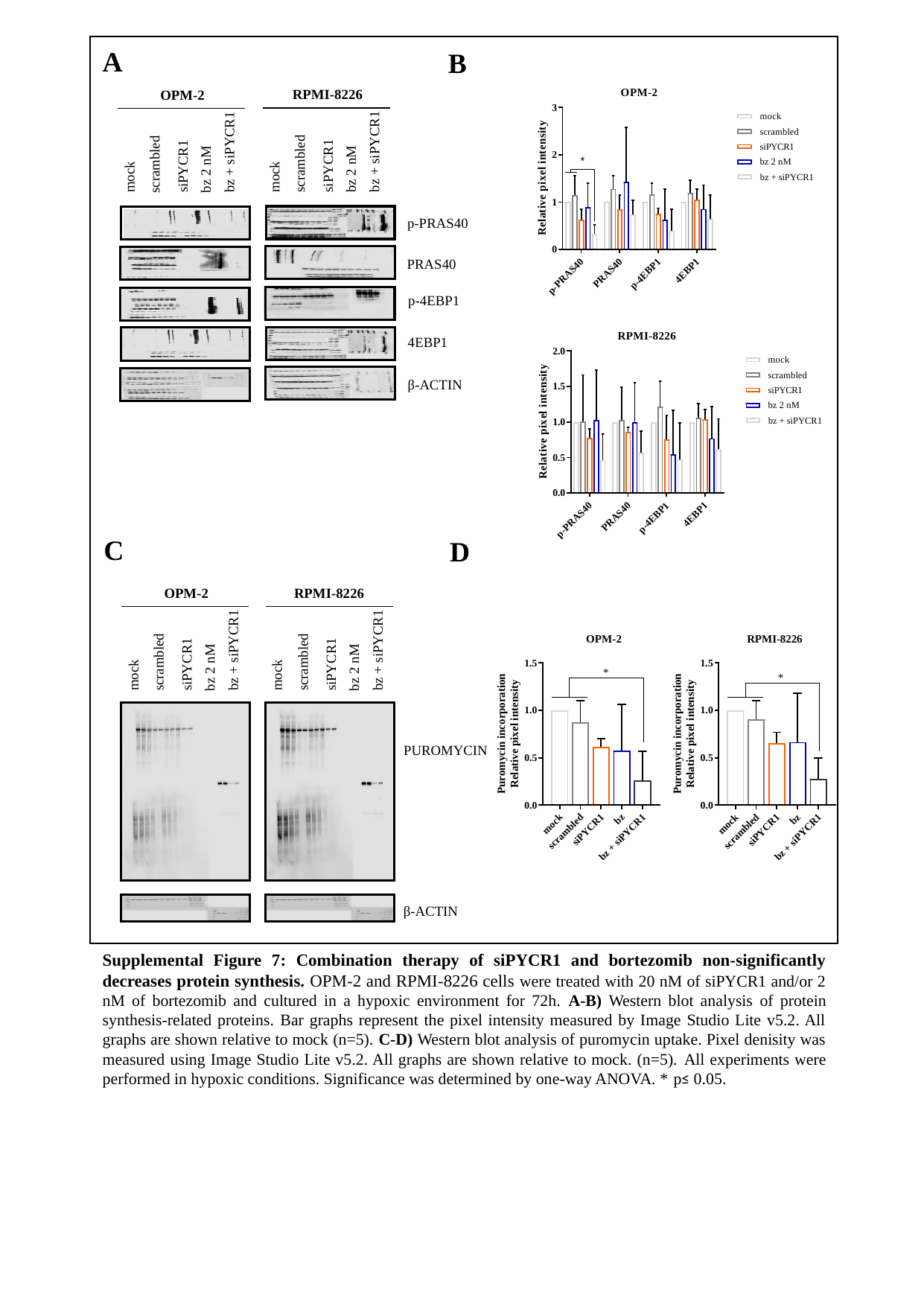

A
B
RPMI-8226
OPM-2
bz + siPYCR1
bz + siPYCR1
scrambled
scrambled
siPYCR1
siPYCR1
bz 2 nM
bz 2 nM
mock
mock
p-PRAS40
PRAS40
p-4EBP1
4EBP1
β-ACTIN
C
D
OPM-2
RPMI-8226
bz + siPYCR1
bz + siPYCR1
scrambled
scrambled
siPYCR1
siPYCR1
bz 2 nM
bz 2 nM
mock
mock
PUROMYCIN
β-ACTIN
Supplemental Figure 7: Combination therapy of siPYCR1 and bortezomib non-significantly decreases protein synthesis. OPM-2 and RPMI-8226 cells were treated with 20 nM of siPYCR1 and/or 2 nM of bortezomib and cultured in a hypoxic environment for 72h. A-B) Western blot analysis of protein synthesis-related proteins. Bar graphs represent the pixel intensity measured by Image Studio Lite v5.2. All graphs are shown relative to mock (n=5). C-D) Western blot analysis of puromycin uptake. Pixel denisity was measured using Image Studio Lite v5.2. All graphs are shown relative to mock. (n=5). All experiments were performed in hypoxic conditions. Significance was determined by one-way ANOVA. * p≤ 0.05.

## Slide 8
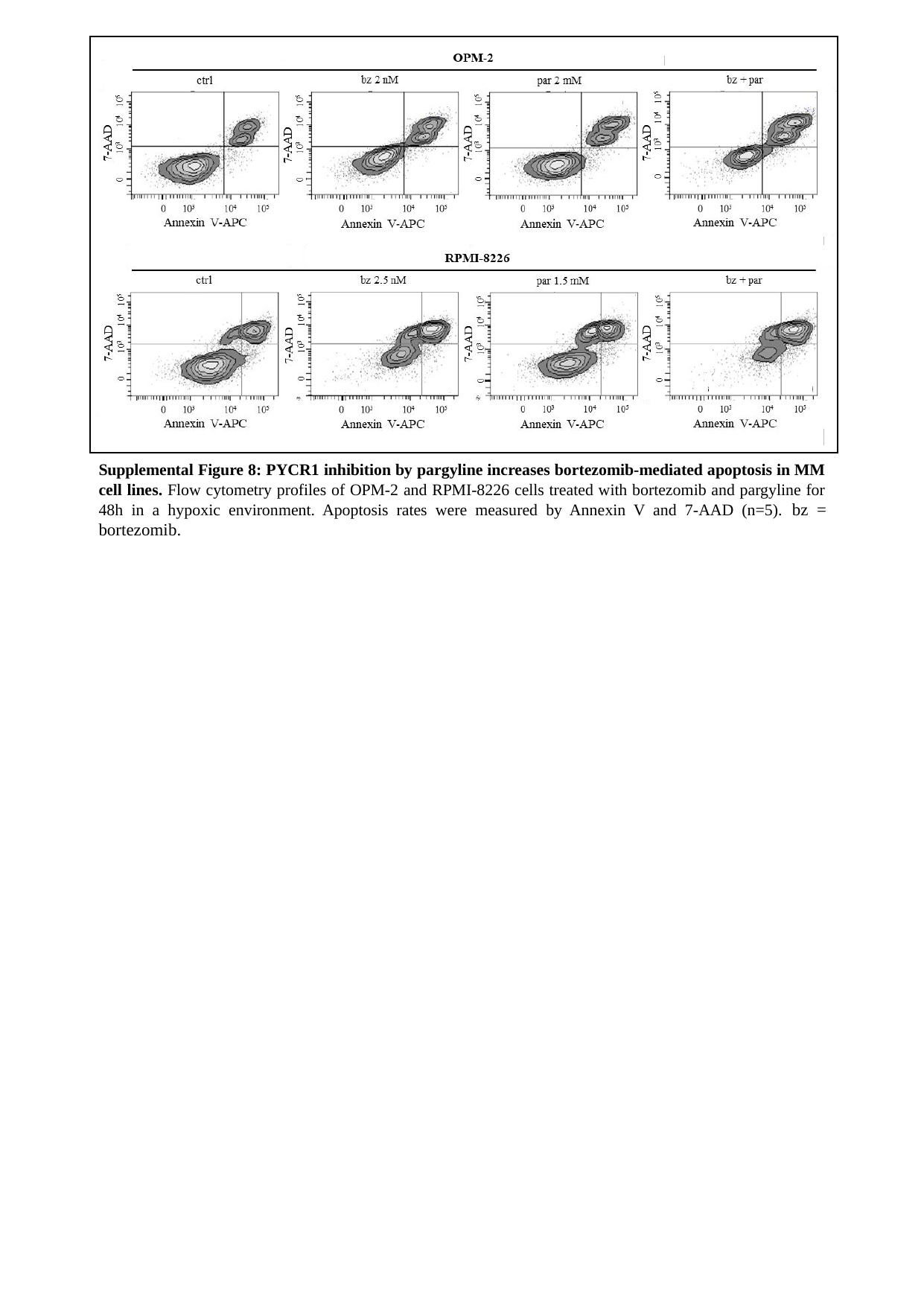

Supplemental Figure 8: PYCR1 inhibition by pargyline increases bortezomib-mediated apoptosis in MM cell lines. Flow cytometry profiles of OPM-2 and RPMI-8226 cells treated with bortezomib and pargyline for 48h in a hypoxic environment. Apoptosis rates were measured by Annexin V and 7-AAD (n=5). bz = bortezomib.

## Slide 9
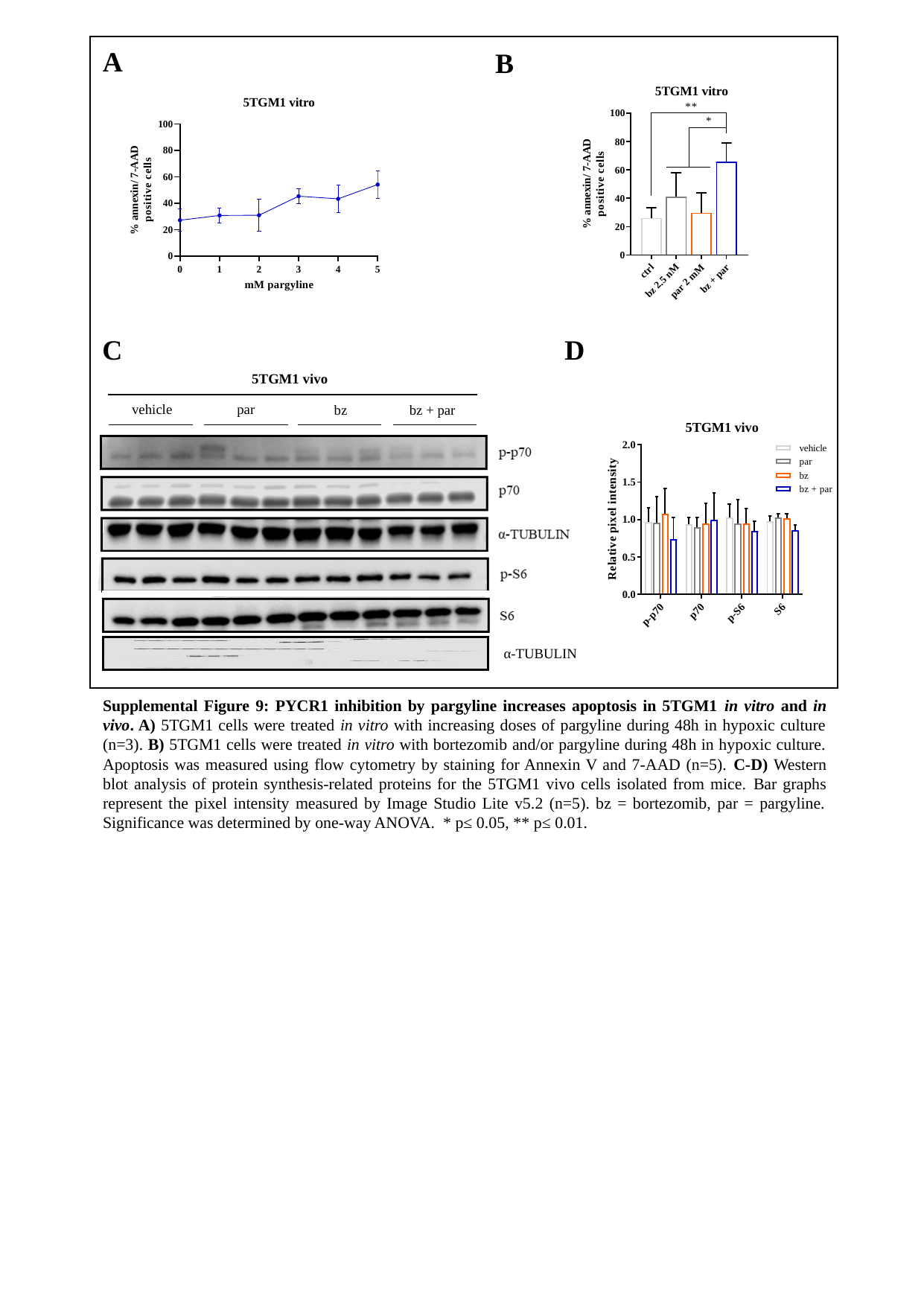

A
B
C
D
5TGM1 vivo
vehicle
par
bz
bz + par
α-TUBULIN
Supplemental Figure 9: PYCR1 inhibition by pargyline increases apoptosis in 5TGM1 in vitro and in vivo. A) 5TGM1 cells were treated in vitro with increasing doses of pargyline during 48h in hypoxic culture (n=3). B) 5TGM1 cells were treated in vitro with bortezomib and/or pargyline during 48h in hypoxic culture. Apoptosis was measured using flow cytometry by staining for Annexin V and 7-AAD (n=5). C-D) Western blot analysis of protein synthesis-related proteins for the 5TGM1 vivo cells isolated from mice. Bar graphs represent the pixel intensity measured by Image Studio Lite v5.2 (n=5). bz = bortezomib, par = pargyline. Significance was determined by one-way ANOVA. * p≤ 0.05, ** p≤ 0.01.

## Slide 10
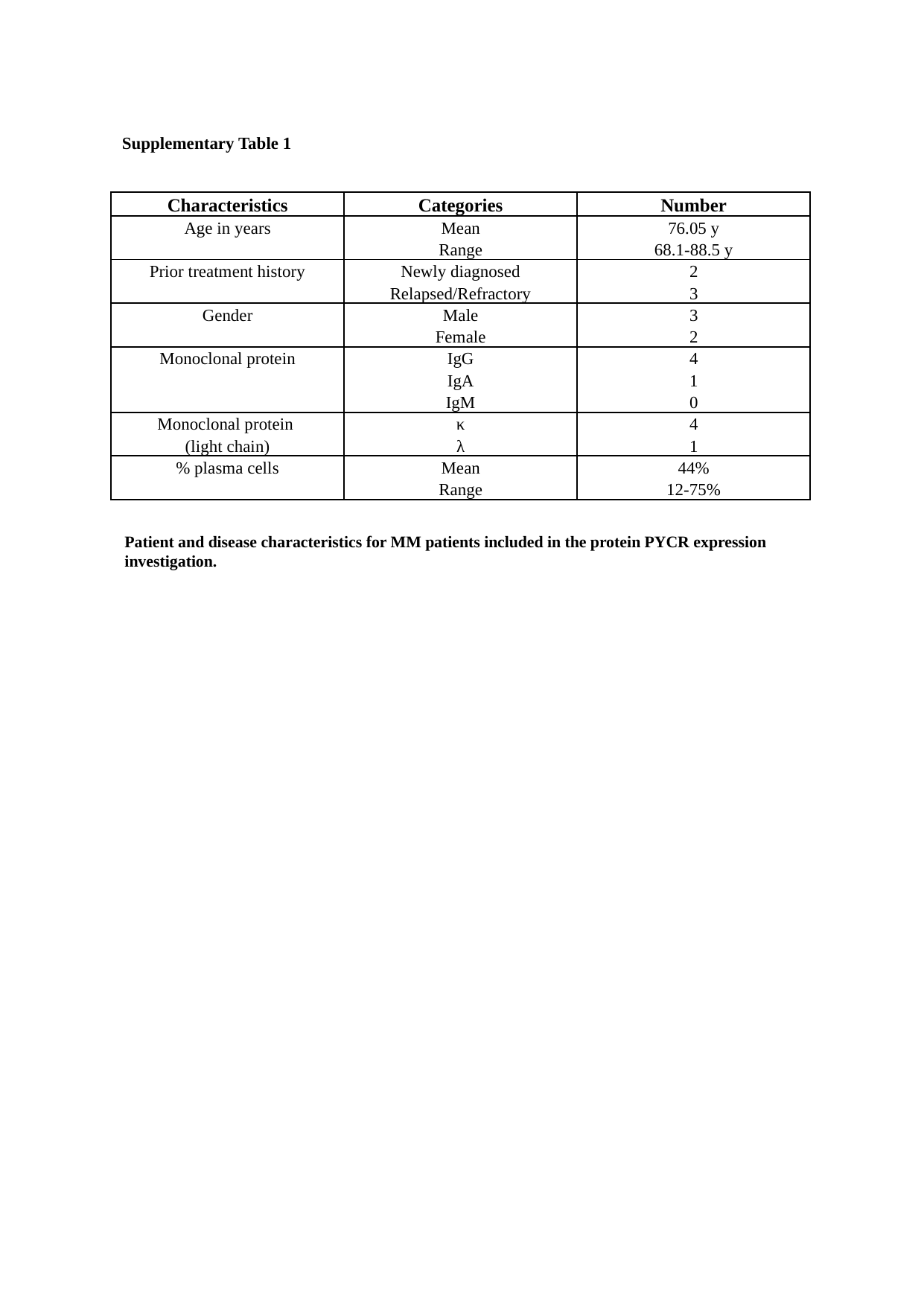

Supplementary Table 1
| Characteristics | Categories | Number |
| --- | --- | --- |
| Age in years | Mean Range | 76.05 y 68.1-88.5 y |
| Prior treatment history | Newly diagnosed Relapsed/Refractory | 2 3 |
| Gender | Male Female | 3 2 |
| Monoclonal protein | IgG IgA IgM | 4 1 0 |
| Monoclonal protein (light chain) | κ λ | 4 1 |
| % plasma cells | Mean Range | 44% 12-75% |
Patient and disease characteristics for MM patients included in the protein PYCR expression investigation.

## Slide 11
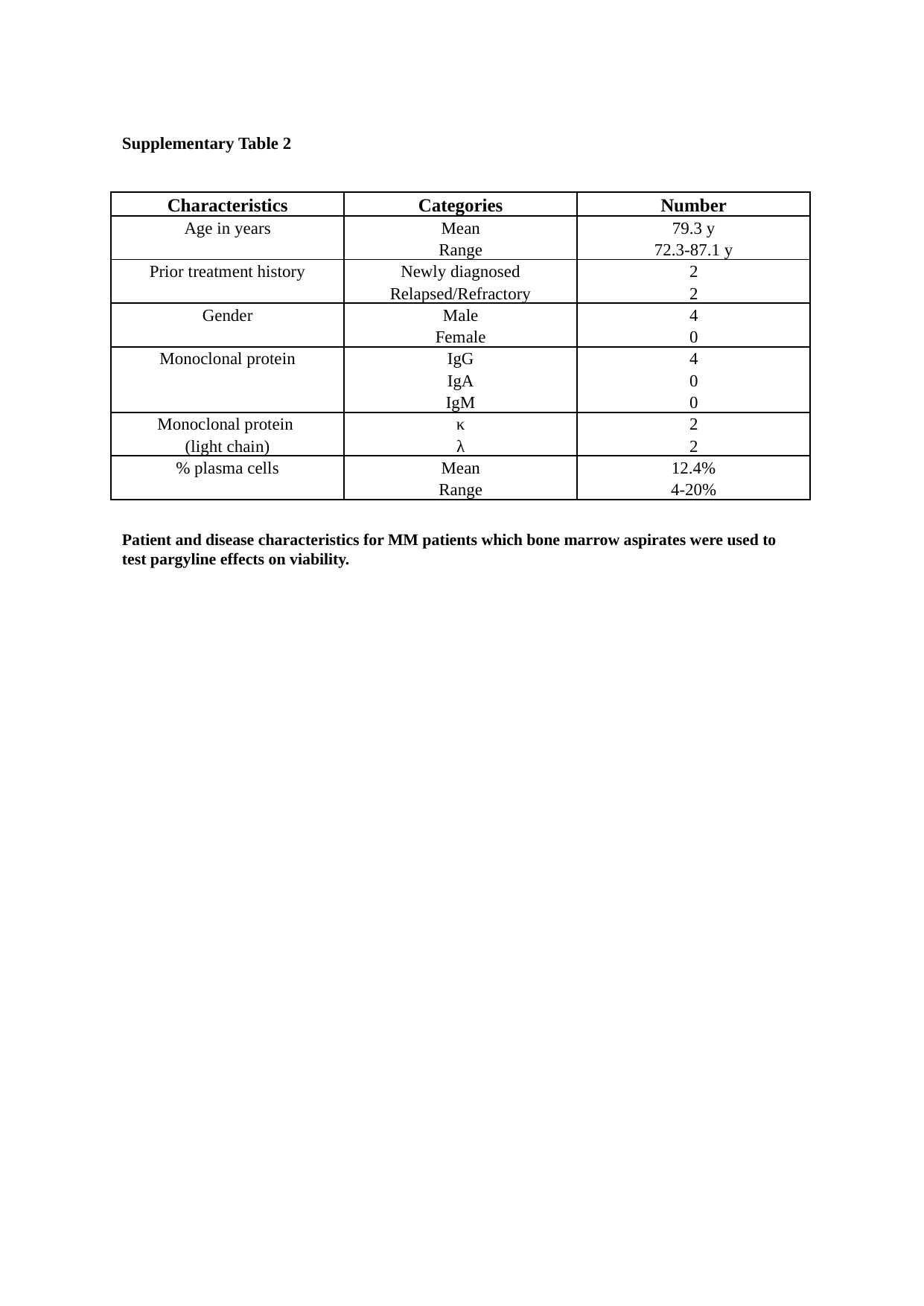

Supplementary Table 2
| Characteristics | Categories | Number |
| --- | --- | --- |
| Age in years | Mean Range | 79.3 y 72.3-87.1 y |
| Prior treatment history | Newly diagnosed Relapsed/Refractory | 2 2 |
| Gender | Male Female | 4 0 |
| Monoclonal protein | IgG IgA IgM | 4 0 0 |
| Monoclonal protein (light chain) | κ λ | 2 2 |
| % plasma cells | Mean Range | 12.4% 4-20% |
Patient and disease characteristics for MM patients which bone marrow aspirates were used to test pargyline effects on viability.
